# Extensive epigenetic reprogramming during the life cycle of *Marchantia polymorpha*

**DOI:** 10.1186/s13059-017-1383-z

**Published:** 2018-01-25

**Authors:** Marc W. Schmid, Alejandro Giraldo-Fonseca, Moritz Rövekamp, Dmitry Smetanin, John L. Bowman, Ueli Grossniklaus

**Affiliations:** 1 Department of Plant and Microbial Biology and Zurich-Basel Plant Science Center, University of Zurich, Zurich, Switzerland; 2 Biological Sciences, Monash University, Clayton, VIC Australia

**Keywords:** Bisulfite sequencing, DNA methylation, Epigenetics, Life cycle, Liverwort, *Marchantia polymorpha*, Reprogramming, Tissue specificity

## Abstract

**Background:**

In plants, the existence and possible role of epigenetic reprogramming has been questioned because of the occurrence of stably inherited epialleles. Evidence suggests that epigenetic reprogramming does occur during land plant reproduction, but there is little consensus on the generality and extent of epigenetic reprogramming in plants. We studied DNA methylation dynamics during the life cycle of the liverwort *Marchantia polymorpha*. We isolated thalli and meristems from male and female gametophytes, archegonia, antherozoids, as well as sporophytes at early and late developmental stages, and compared their DNA methylation profiles.

**Results:**

Of all cytosines tested for differential DNA methylation, 42% vary significantly in their methylation pattern throughout the life cycle. However, the differences are limited to few comparisons between specific stages of the life cycle and suggest four major epigenetic states specific to sporophytes, vegetative gametophytes, antherozoids, and archegonia. Further analyses indicated clear differences in the mechanisms underlying reprogramming in the gametophytic and sporophytic generations, which are paralleled by differences in the expression of genes involved in DNA methylation. Differentially methylated cytosines with a gain in methylation in antherozoids and archegonia are enriched in the CG and CHG contexts, as well as in gene bodies and gene flanking regions. In contrast, gain of DNA methylation during sporophyte development is mostly limited to the CHH context, LTR retrotransposons, DNA transposons, and repeats.

**Conclusion:**

We conclude that epigenetic reprogramming occurs at least twice during the life cycle of *M. polymorpha* and that the underlying mechanisms are likely different between the two events.

**Electronic supplementary material:**

The online version of this article (doi:10.1186/s13059-017-1383-z) contains supplementary material, which is available to authorized users.

## Background

Epigenetic reprogramming refers to global changes in DNA methylation and histone modifications (together referred to as the epigenome) between different stages of development. For instance, the erasure of epigenetic marks between one generation and the next removes modifications that accumulated during the lifetime of an organism and sets the stage for zygotic development. Thus, epigenetic reprogramming results in the existence of epigenomes that are specific to different stages of the life cycle, and which are not inherited from the previous stage, but actively set during transition from one stage to the next. As a consequence, the epigenomes of two different individuals at the same developmental stage are more similar to each other than the epigenomes of one individual at two different developmental stages. Compared to histone modifications, DNA methylation is more accessible [1], such that whole-genome DNA methylation profiling has been the method of choice to study epigenetic reprogramming [2]. In mammals, epigenetic reprogramming is associated with sexual reproduction and occurs in two major waves: during primordial germ cell formation and in the zygote. The reprogramming comprises an almost complete erasure of DNA methylation marks, followed by their re-establishment [3, 4]. Given the extensive resetting of epigenetic marks, transgenerational inheritance of epigenetic variants (epialleles) is thought to be rare in mammals [5, 6].

In contrast to mammals, land plants do not have a predefined germline and follow a more complex life cycle with an alternation between two heteromorphic and multicellular generations: the diploid sporophyte and the haploid gametophyte [7]. In the sporophyte, distinct cells undergo meiosis and produce spores. These give rise to multicellular gametophytes, which produce the male and female gametes through mitotic divisions. Fusion of a male gamete (sperm cell or antherozoid) and a female gamete (egg cell) results in a zygote, which forms the sporophyte of the next generation. Thus, the germline is not set aside early during development but forms only later when somatic cells are committed to form gametes. Epigenetic marks gained during development or induced by environmental conditions are thus potentially heritable. Indeed, there are several examples of stably inherited epialleles in plants [8, 9] and their existence led to the hypothesis that epigenetic reprogramming might not exist in plants [2]. However, recent studies provide direct or indirect evidence for dynamic changes in histone modifications during sporogenesis [10], DNA methylation during gametogenesis [11–15], and DNA methylation during embryogenesis [16]. Thus, epigenetic reprogramming does also occur in plants, at least to a certain extent [2, 10, 17, 18]. However, these studies focused on either male or female gametogenesis or embryogenesis of flowering plants and do not provide a comprehensive view on the entire life cycle. Thus, there currently seems to be little consensus on the overall extent of epigenetic reprogramming in plants [2, 9]. Lastly, considering the different modes of sexual reproduction and different overall patterns of DNA methylation across the plant kingdom [19], it is likely that there are also differences in epigenetic reprogramming between species.

**Fig. 1:**
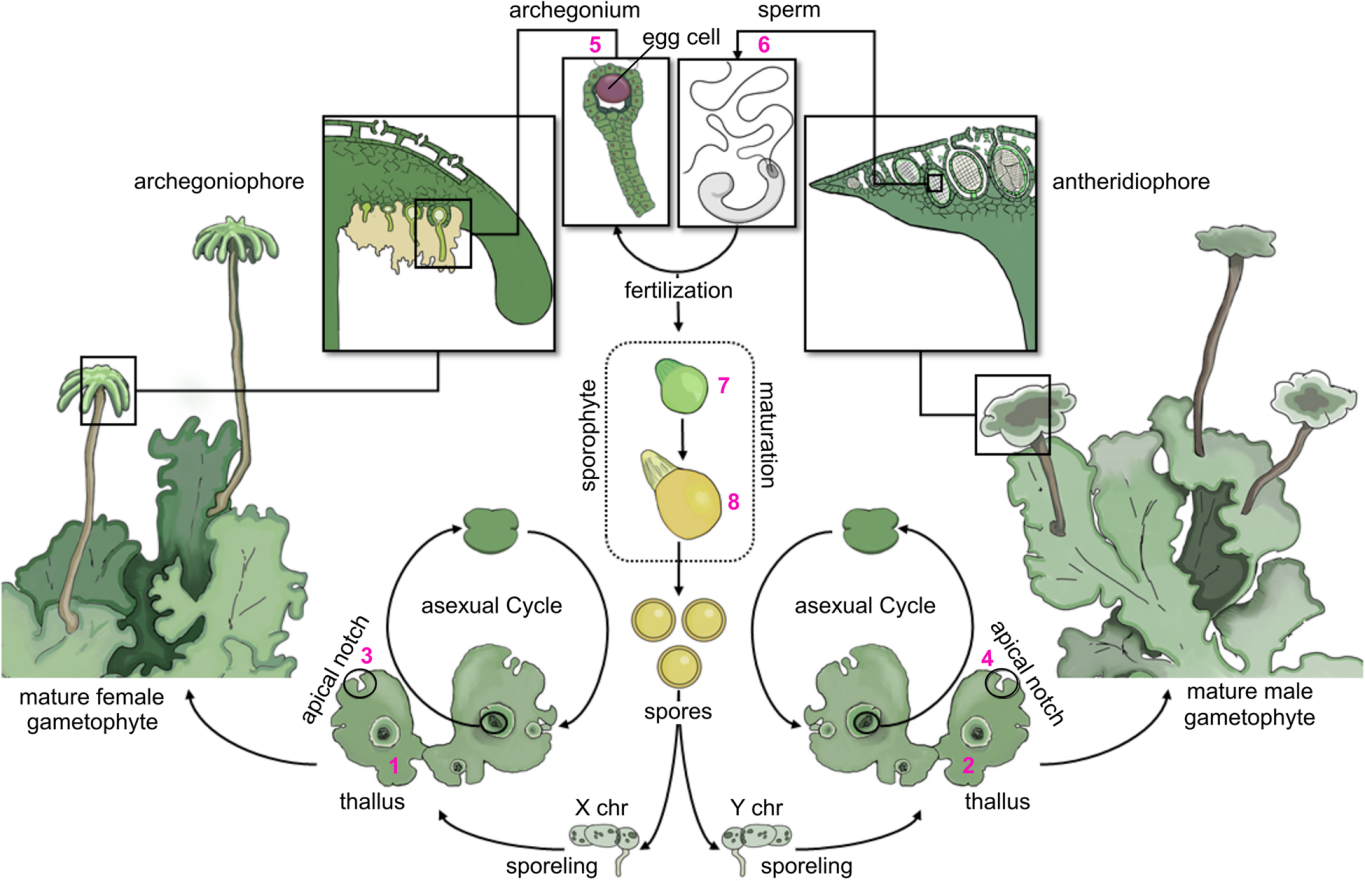
*Illustration* of the life cycle of *M. polymorpha*. Land plants have a more complex life cycle than animals, alternating between two multicellular, heteromorphic generations: the sporophyte and the gametophyte. In bryophytes (mosses, hornworts, and liverworts), the gametophytes constitute the dominant generation while the sporophyte is a small and simple structure, whose development depends on the female gametophyte. The male gametophyte forms antheridiophores, the reproductive structures that harbor the antheridia with the antherozoids (sperm). The female gametophyte forms archegoniophores, the reproductive structures that harbor the archegonia, each of which contains a single egg cell. During sexual reproduction, the antherozoids are released from the antheridia and swim towards the archegonia on the female gametophyte. The sporophyte is formed upon fertilization and remains attached to the archegoniophore during its entire development. Spores are formed in the sporophyte through meiosis and are finally released. The spores germinate and develop into either male (with Y chromosome) or female (with X chromosome) gametophytes, thereby concluding the life cycle. Both, male and female gametophytes are capable of asexual reproduction through the formation of gemmae in the gemma cups [57]. Numbers in *magenta* mark the tissues isolated for this study: (1/2) thallus of the female/male gametophyte, respectively, (3/4) apical notch of the female/male gametophyte, respectively, (5) archegonia, (6) antherozoids, (7) early sporophyte, and (8) late sporophyte

To contribute to the understanding of the extent of epigenetic reprogramming in plants, we studied the DNA methylation dynamics during the life cycle of the liverwort *Marchantia polymorpha*, a member of the probably most basal lineage of extant land plants [20, 21]. Due to its phylogenetic context, simple life cycle, small genome size, the absence of evidence for ancient polyploidization, and the lack of gene duplication, *M. polymorpha* has recently received increasing attention as a model organism [22–24]. In contrast to flowering plants, the gametophyte of *M. polymorpha* represents the dominant generation, while the sporophyte is a small and ephemeral structure that completely depends on the female gametophyte for its development (Fig. 1). Recent efforts to sequence and annotate the genome of *M. polymorpha* have further revealed that, based on the genes known in the model plant *Arabidopsis thaliana*, it contains a complete DNA methylation machinery [25]. The genome of *M. polymorpha* codes for five DNA methyltransferases belonging to three different classes [25]: the DNA METHYLTRANSFERASE family protein MpMET (maintains methylation in CG context [2]); the plant-specific CHROMOMETHYLASE family proteins MpCMTa and MpCMTb (de novo methylation in CHG and CHH contexts [2]); and the DOMAINS REARRANGED METHYLTRANSFERASE family proteins MpDRMa and MpDRMb (de novo methylation in all contexts, including CHG and CHH sites not methylated by CMT [2]). Given its phylogenetic position, the low complexity of its genome, and the presence of maintenance and de novo methyltransferases, *M. polymorpha* is an attractive model system to study DNA methylation dynamics throughout its life cycle.

By sampling eight tissues from males and females, we provide the most comprehensive dataset on tissue-specific methylation in plants to date and show that 42% of all assessed cytosines vary significantly in their methylation pattern throughout the *M. polymorpha* life cycle. We identified four distinct epigenetic landscapes among these tissues and show that epigenetic reprogramming occurs at least twice, once in both the gametophytic and sporophytic generation, with each event relying on a distinct mechanism.

## Results and discussion

### Overall DNA methylation levels show tissue-specific differences in *M. polymorpha*

To date, very little tissue-specific information on genome-wide DNA methylation is available in plants [16, 26–29] and cell type-specific data are largely restricted to *A. thaliana* gametes [12–14] and root cells [30]. To characterize the DNA methylation dynamics during the life cycle of *M. polymorpha*, we isolated thalli and apical notches (i.e. gametophytic meristems) from male and female gametophytes, archegonia (gametangia containing the egg cells), antherozoids (sperm), and sporophytes at two developmental stages (Fig. 1). We isolated all samples from the same three male and female individuals (gametophytic tissues, archegonia, and antherozoids) and three pairwise crosses between these individuals (sporophytes), resulting in three biological replicates per tissue type. Genome-wide DNA methylation levels were determined by whole-genome bisulfite sequencing (WGBS). Cytosines with a total read coverage < 5 or > 100 were excluded from all subsequent analyses to avoid a potential bias originating from low coverage or poorly annotated sequences [31]. On average, 14 million cytosines (24%) passed this filter per sample (see Additional file 1: Table S1). DNA methylation levels for a given cytosine were calculated as the percentage of the coverage, indicating methylation compared to the total coverage.

**Fig. 2:**
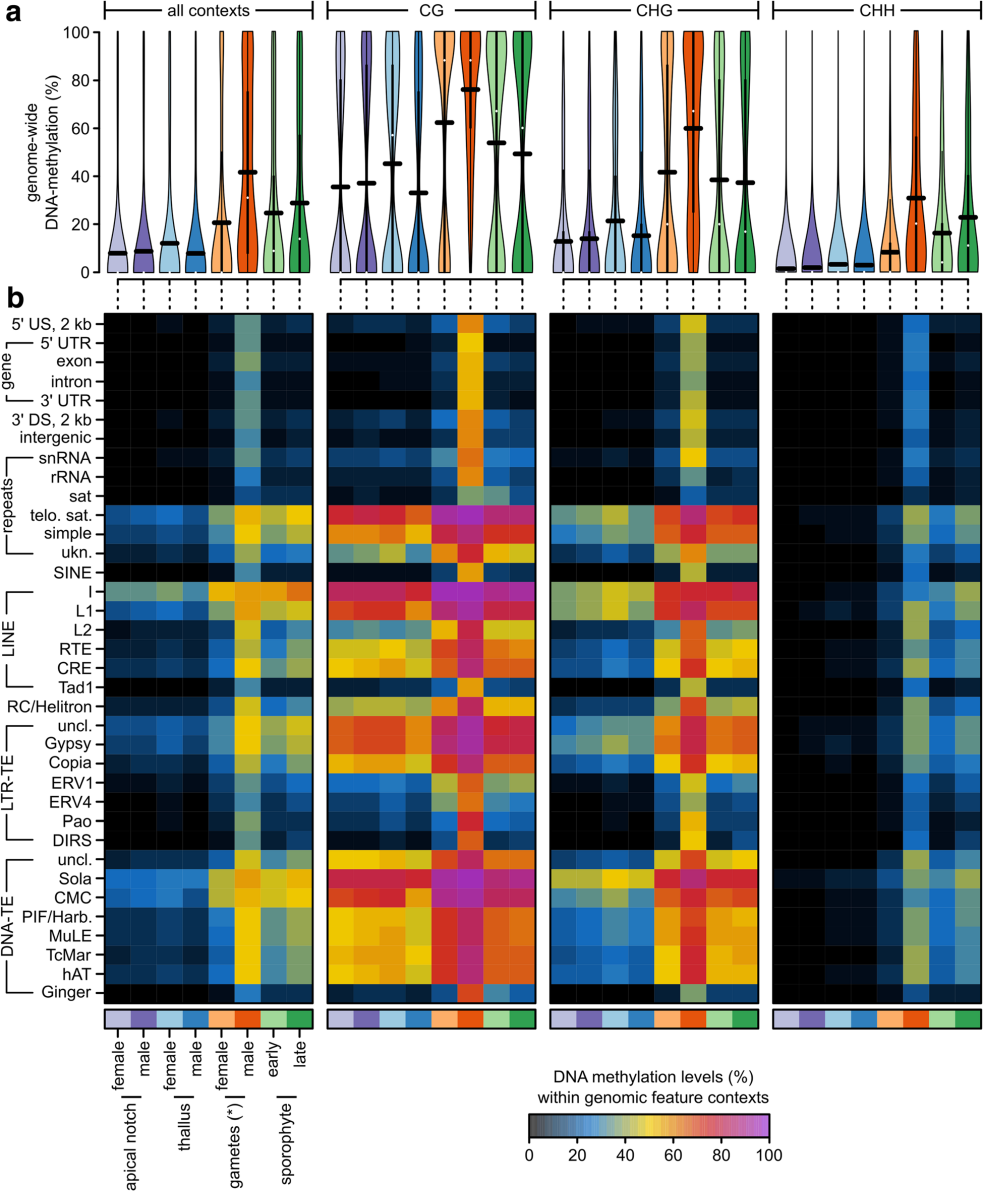
**a** DNA methylation levels in percent at individual cytosines located in autosomes across all or within each individual sequence context (CG, CHG, CHH) for each tissue type used in this study shown as *violin plots* (see Additional file 1: Figure S1 for data from individual replicates). The *horizontal black bars* correspond to the means (see Additional file 1: Tables S2 and S3). **b** Average DNA methylation levels in percent for each sequence context, genomic feature, and tissue type shown as a *heatmap* (see Additional file 1: Table S4, S5, S6, and S7). US/DS upstream/downstream of a gene, UTR untranslated region, snRNA small nucleolar RNA, rRNA ribosomal RNA, sat satellite repeat, telo. sat telomeric satellite repeats, simple simple repeats, ukn. unknown/unclassified repeats, SINE short interspersed nuclear elements, LINE long interspersed nuclear elements, RC rolling circle transposon, LTR-TE retrotransposon with long terminal repeats, DNA-TE DNA transposon, uncl. not classified into a subfamily. *Note that for the “female gametes,” we sampled archegonia, which are entire gametangia that harbor the egg cells

In plants, DNA cytosine methylation occurs in three different contexts: CG, CHG, and CHH (where H stands for A, T, or C). DNA methylation levels generally vary between these contexts and differ between genomic regions [19]. To get a genome-wide overview of DNA methylation levels in the different sequence contexts, we first compared the overall levels of DNA methylation in the different tissue types with an ANOVA (Fig. 2a, see Additional file 1: Table S2 and S3). DNA methylation levels were on average lowest in the CHH (11.16%), higher in the CHG (28.82%), and highest in the CG context (46.40%, *P*_*context*_ < 0.0001, explained 49.03% of all variation). This trend depended on the tissue type (*P*_*context:tissue*_ < 0.0001, explained 4.66% of all variation), but methylation was always highest in CG and lowest in CHH context. Irrespective of the context, overall methylation levels varied greatly between the tissue types (*P*_*tissue*_ < 0.0001, explained 43.24% of all variation). DNA methylation was generally lowest in thalli and apical notches (15–23%), higher in archegonia and sporophytes (34–37%), and highest in antherozoids (55.16%; Fig. 2a, see Additional file 1: Tables S2 and S3).

We then extended the ANOVA by including the different genomic features (Fig. 2b, see Additional file 1: Tables S4–S7, all *P* values < 0.0001, except for *P*_*context:tissue:feature*_ > 0.99). Overall, the genomic features explained around 44.1% of all variation (sequence context = 25.9%, tissue type = 21.3%). The pairwise interactions were significant, but contributed little to overall variation (1.9–2.7%). Thus, differences in DNA methylation were largely driven by the genomic feature context, the sequence context, and the tissue type. Averaged across the different sequence contexts (Fig. 2b, see Additional file 1: Table S4), DNA methylation was highest in long interspersed nuclear elements (LINEs, up to 45.29%), DNA transposons (up to 38.02%), telomeric satellite repeats (32.98%), LTR retrotransposons (up to 30.09%), and simple repeats (28.04%). In contrast, DNA methylation was low in genes (8.26% in exons, 6.20% in introns), their flanking regions (each around 9%), ribosomal RNA (7.41%), and satellite repeats (6.56%). The DNA methylation enrichment in repetitive elements and the depletion in gene bodies was overall consistent with a previous report using data from only vegetative gametophytic tissues [19]. However, differences between the tissue types reported above (antherozoids > sporophytes and archegonia > gametophytes) were largely consistent across the different genomic features. For example, DNA methylation was almost always highest in antherozoids irrespective of any sequence and genomic feature context (Fig. 2b, see Additional file 1: Table S5–S7). As a consequence, DNA methylation in antherozoids within gene bodies (exons) reached 54.00% in the CG, 41.42% in the CHG, and 27.54% in the CHH context. Thus, unlike previously reported [19], *M. polymorpha* does not generally lack DNA methylation in gene bodies. Instead, it restricts it to specific stages of the life cycle.

DNA methylation in gene bodies (exons) in the archegonia (gametangia containing the egg cells) were 14.38% in the CG, 10.36% in the CHG, and 5.19% in the CHH context and thus slightly increased compared to the gametophytes (~4.4%/~ 2.4%/~ 1.0% in the CG/CHG/CHH context, respectively). However, because the archegonia contain both, gametophytic tissues and egg cells, it was not possible to distinguish whether the differences between the archegonia and the antherozoids originate from differences between the egg cells and the antherozoids or if the differences were caused by the dilution of the egg cells with the gametophytic tissue of the archegonia.

### Sex chromosomes and autosomes show distinct DNA methylation patterns

**Fig. 3:**
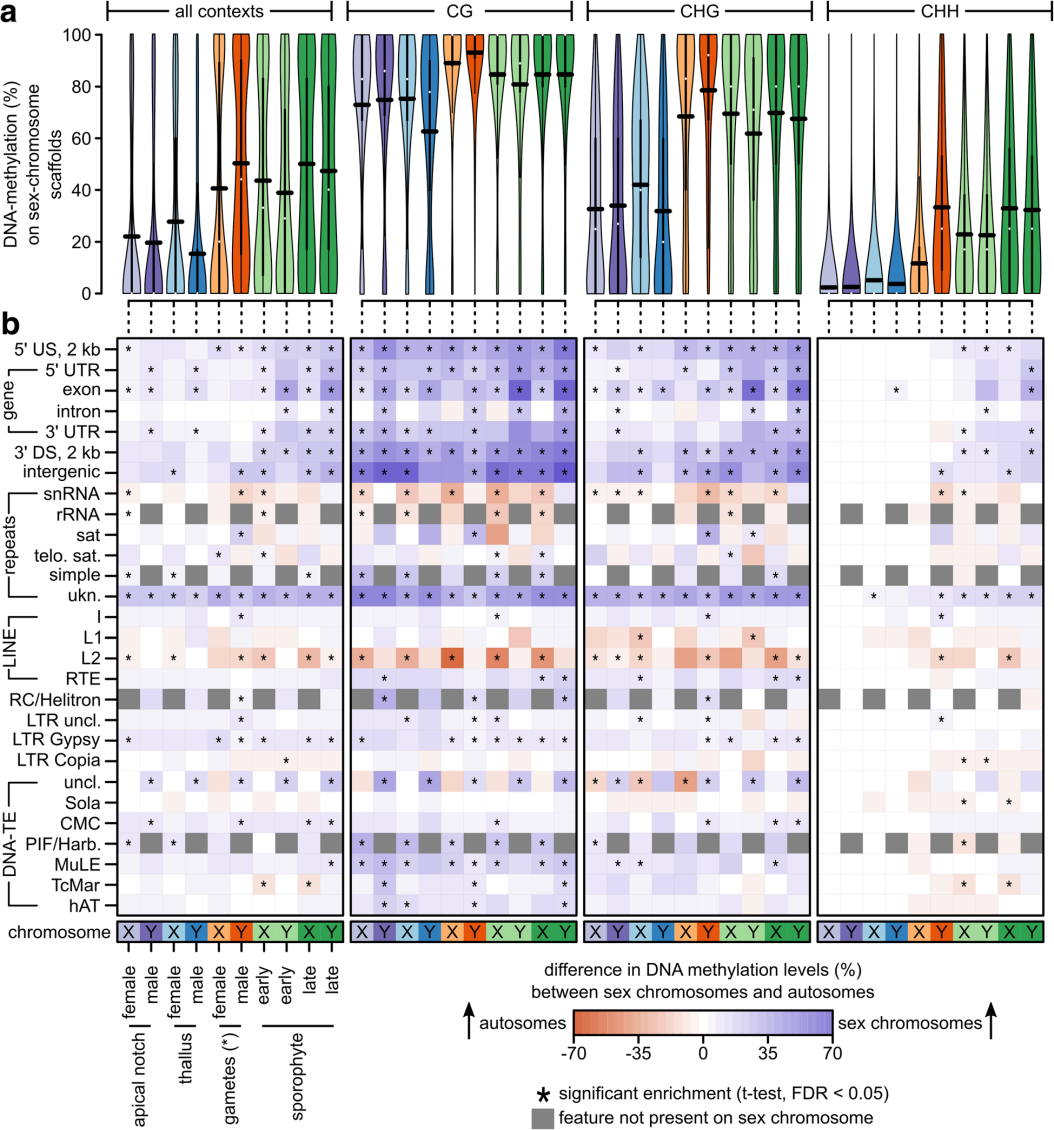
**a** DNA methylation levels in percent at individual cytosines located in sex chromosomes across all or within each individual sequence context (CG, CHG, CHH) for each tissue type used in this study shown as *violin plots*. The *horizontal black bars* correspond to the means (see Additional file 1: Table S9). Female/male gametophytes and gametes only contain the X/Y chromosome. Sporophytes contain both, X and Y, sex chromosomes. **b** Difference in the average DNA methylation level between the individual sex chromosomes and the autosomes for each sequence and genomic feature context. Fields marked with an *asterisk* depict comparisons that were statistically significant (two-sided t-test, adjusted for multiple testing, FDR < 0.05). *Gray fields* depict cases in which the given genomic feature is not present on the sex chromosome. US/DS upstream/downstream of a gene, UTR untranslated region, snRNA small nucleolar RNA, rRNA ribosomal RNA, sat satellite repeat, telo. sat telomeric satellite repeats, simple simple repeats, ukn. unknown/unclassified repeats, SINE short interspersed nuclear elements, LINE long interspersed nuclear elements, RC rolling circle transposon, LTR-TE retrotransposon with long terminal repeats, DNA-TE DNA transposon, uncl. not classified into a subfamily. *Note that for the “female gametes,” we sampled archegonia, which are entire gametangia that harbor the egg cells

We investigated whether sex chromosomes (Y and X chromosomes in males and females, respectively) and autosomes differ in their methylation patterns. Because the *M. polymorpha* genome is not assembled into chromosomes and consists of 2957 scaffolds, we compared cytosine methylation between the autosomal scaffolds, the two scaffolds making up the Y chromosome, and the nine scaffolds constituting the X chromosome [25]. Overall DNA methylation levels in sex chromosomes (80.20/55.48/16.88% in the CG/CHG/CHH context, respectively; Fig. 3a, see Additional file 1: Table S9) were clearly higher than in autosomes (46.40/28.82/11.16% in the CG/CHG/CHH context, respectively; Fig. 2b, see Additional file 1: Table S2). To normalize for differences between different sequence contexts, genomic feature contexts, and tissue types, we tested within each combination whether there was a difference in the average DNA methylation level (in percent) between the sex chromosome(s) and autosomes (Fig. 3b, two-sided t-test, corrected for multiple testing, false discovery rate [FDR] < 0.05). In the CG context, DNA methylation levels of sex chromosomes were significantly increased within gene bodies and their flanking regions, within unknown types of repeats, within LTR retrotransposons of the Gypsy group (only some tissues), and some DNA transposons (MuLE and PIF/Harbinger). In a few cases, there were differences between the X and the Y chromosome. For example, DNA methylation levels in introns were increased in the Y chromosome, but not the X chromosome. Likewise, exon methylation levels in sporophytes were more increased in the Y chromosomes than the X chromosome (on average, 41/40/24% more methylation increase in the CG/CHG/CHH contexts on the Y compared to the X chromosome, two-sided t-test, all *P* < 0.02). This pattern was similar but weaker in the CHG context, and much weaker in the CHH context. In the latter, differences were almost exclusively found in the sporophytes, which carry both sex chromosomes. The general increase in gene body DNA methylation in all tissue types could be explained by a spreading of DNA methylation from nearby transposons and repeats, which make up around 70% of the sex chromosomes [25, 32]. However, it is unclear whether this alone could explain the even more pronounced increase in gene body DNA methylation in sporophytes (especially on the Y chromosome), in which the two sex chromosomes co-occur. We hypothesized that imprinting or gene dosage regulation could result in increased DNA methylation in genes that are shared between the sex chromosomes. Thus, we expected genes shared between sex chromosomes to exhibit the largest increase in DNA methylation in sporophytes compared to gametophytes.

To investigate whether dosage compensation of genes shared between the sex chromosomes caused the differences between the X and Y chromosomes in sporophytes, we visualized the differences in average DNA methylation levels in sex chromosome genes (exons and flanking regions) between the sex chromosomes in the sporophytes and the sex chromosomes in the gametophytes (see Additional file 1: Figure S2). We then sorted the average differences and inspected whether genes shared between the sex chromosomes [25] were among the ones with the largest increase of DNA methylation. Overall, most genes showed an increase in DNA methylation in the sporophytes compared to the gametophytes. However, in contrast to the hypothesis, genes shared between the sex chromosomes were not among the top, but rather among the genes with the weakest increase in DNA methylation. It is therefore unlikely that dosage compensation of shared genes caused the clear increase in gene body DNA methylation in the sex chromosomes of sporophytes. Thus, it may rather be a general differential regulation of sex chromosome genes in the sporophyte, which results in the more pronounced enrichment of DNA methylation in the sporophytic sex chromosomes compared to the sporophytic autosomes.

### Four distinct epigenetic landscapes are found among *M. polymorpha* tissues

As shown above, genome-wide DNA methylation patterns were clearly distinct between the different tissue types. However, to identify the extent of epigenetic reprogramming, it is necessary to identify epigenomes that are specific to the different stages of the life cycle, i.e. there must be cytosines that are consistently more methylated in one stage compared to the other. Also, if epigenetic reprogramming is extensive, there should be few cytosines that are specific to a given individual and invariant between the different stages of the life cycle of the given individual. We therefore tested for differential cytosine methylation between the different tissue types and sexes, as well as between different individuals.

To this aim, we extracted all cytosines for which there were data available from at least two out of three replicates from each tissue (sex chromosomes were excluded because they are not shared between all tissue types). We analyzed the variation at each individual cytosine with a linear model and compared specific groups of interests to each other with linear contrasts. The advantage of this approach, compared to multiple pairwise comparisons, is that each individual comparison is based on the same data and the same residual estimates. The results of the different comparisons are therefore more comparable to each other than a set of pairwise comparisons (see “Methods” for details). *P* values for each comparison were adjusted for multiple testing to reflect FDRs and a cytosine was defined as differentially methylated (DMC) if the FDR was < 0.001. In total, we tested around 1 million cytosines for which there were enough data available. Compared to all cytosines in the *M. polymorpha* genome and random subsets of cytosines with identical context frequencies, these positions exhibited enrichment in the CHH context and transposable elements (TEs; *P* < 0.01; see Additional file 1: Figure S3). It is therefore important to note that conclusions drawn from this analysis are slightly biased towards the CHH context and transposons.

To estimate the fraction of cytosines with a DNA methylation level specific to a certain individual, we compared all individuals to each other using all haploid tissues of the individuals as replicates (i.e. everything except the sporophytes). On average, we found 373/6/5 DMCs in the CG/CHG/CHH context, respectively, between two different individuals. This corresponded to 0.3%, 0.005%, and 0.0007% of all tested cytosines (see Additional file 1: Table S10). Thus, DNA methylation levels at individual cytosines were almost never consistently inherited across the life cycle.

**Fig. 4:**
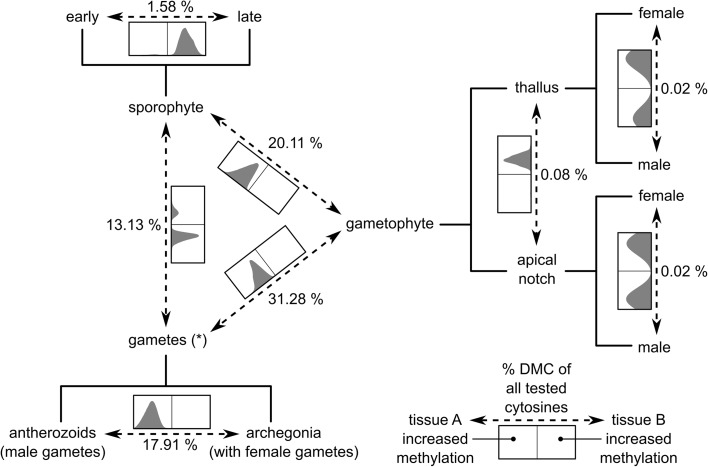
*Schematic representation* of the comparisons performed during the analysis of differential cytosine methylation. Variation in DNA methylation at each individual cytosine was analyzed with a linear model according to a design with a single factor comprising all different experimental groups [14]. Specific groups were then compared with linear contrasts. Percentages of cytosines with significant (FDR < 0.001) differences in DNA methylation are given for each comparison (total number of cytosines tested: 994,696). In addition, there is a histogram with the differences in DNA methylation at the individual DMCs for each comparison (the sizes are not proportional to the number of DMCs found in the comparison). The *x-axis* of the histogram ranges from – 100% to + 100% and the *vertical line* is at 0. The orientation of the *x-axis* is such that a higher density in the *left*/*right* side of the *histogram* corresponds to higher methylation in the group at the *left*/*right* side of the *arrow* depicting the comparison. For example, almost all DMCs identified in the comparison “gametophyte vs sporophyte” have increased methylation levels in the sporophytes compared to the gametophytes. See Additional file: Figure S4 for differences in DNA methylation for each individual sequence and genomic feature context

To determine the cytosines with DNA methylation specific to tissue types or sexes, we compared: (1) gametangia/gametes (archegonia and antherozoids) vs gametophytes (thalli, apical notches); (2) sporophytes vs gametophytes; (3) gametangia/gametes vs sporophytes; (4) antherozoids vs archegonia; (5) late vs early sporophytic tissue; (6) fully differentiated thallus (thalli) vs meristematic thallus tissue (apical notches); (7) male vs female thalli; and (8) male vs female apical notches (Fig. 4, see Additional file 1: Figure S4). Out of the approximately 1 million cytosines, around 42% exhibited significant differential methylation in at least one of the comparisons (413,059 DMCs). However, the number of DMCs identified in the individual comparisons varied greatly. There were almost no significant differences between differentiated thalli and meristematic apical notches (789 DMCs, 0.08% of all tested) or male and female thalli or apical notches (115 and 135 DMCs, respectively; each 0.01% of all tested). This is consistent with the fact that there are no morphological or anatomical sexual dimorphisms in the thalli of this species and that (nearly) every cell is capable of regenerating another thallus and, thus, retains its totipotent potential.

In contrast, we could identify 311,132 DMCs (31.28%) between gametangia/gametes (archegonia and antherozoids) and gametophytes, 200,001 DMCs (20.11%) between sporophytes and gametophytes, 130,583 DMCs (13.13%) between gametangia/gametes and sporophytes, 178,165 DMCs (17.91%) between antherozoids and archegonia, and 15,751 DMCs (1.58%) between early and late sporophytic tissues. Interestingly, most of the comparisons were strongly biased towards one side having a high level of methylation compared to the other (Fig. 4, see Additional file 1: Figure S4). For example, almost all DMCs (99.98%) between gametangia/gametes and gametophytes had increased methylation in the gametangia/gametes. Likewise, almost all DMCs (99.87%) between sporophytes and gametophytes had increased methylation in the sporophytes. In a similar way, DNA methylation was mostly higher in late compared to early sporophytes (98.74%) and in antherozoids compared to archegonia (99.96%). Only in the comparison between gametangia/gametes and sporophytes, both groups contained DMCs with higher levels of DNA methylation (78.50% in gametangia/gametes, 21.50% in sporophytes). Overall, DMCs of all comparisons were most often found in LTR retrotransposons (Copia and Gypsy), unknown types of repeats, genes and their flanking regions, LINEs (RTE), and DNA transposons (hAT; see Additional file 1: Figure S4).

Taken together, these results suggest the existence of four major epigenetic landscapes, which are specific to sporophytes, vegetative gametophytic tissues, antherozoids, and archegonia. DNA methylation levels are generally low in the gametophytes and differences between tissue types were largely driven by a gain of DNA methylation in antherozoids and archegonia, a loss in (early) sporophytes compared to the gametangia/gametes, and another gain during sporophyte development.

### Distinct mechanisms of epigenetic reprogramming occur during reproductive and sporophytic development of *M. polymorpha*

To test whether there is complete reprogramming of the DNA methylation pattern, it would be best to track DNA methylation levels of all cytosines over several generations. However, this would require a higher sequencing coverage (saturation) to evaluate the entire genome and more generations. Given the limited sequencing coverage and the single generation analyzed, we instead focused on specific subsets of cytosines that we consider strong indicators of epigenetic reprogramming. Given that a gain of DNA methylation is always an active process (as opposed to loss of DNA methylation which can occur passively through cell division), we focused on DMCs that clearly gained methylation in: (1) antherozoids or archegonia compared to vegetative gametophytic tissues (309,834 DMCs); (2) early sporophytes compared to gametangia/gametes (24,073 DMCs); and (3) during sporophyte development from early to late stages (15,549 DMCs).

**Fig. 5:**
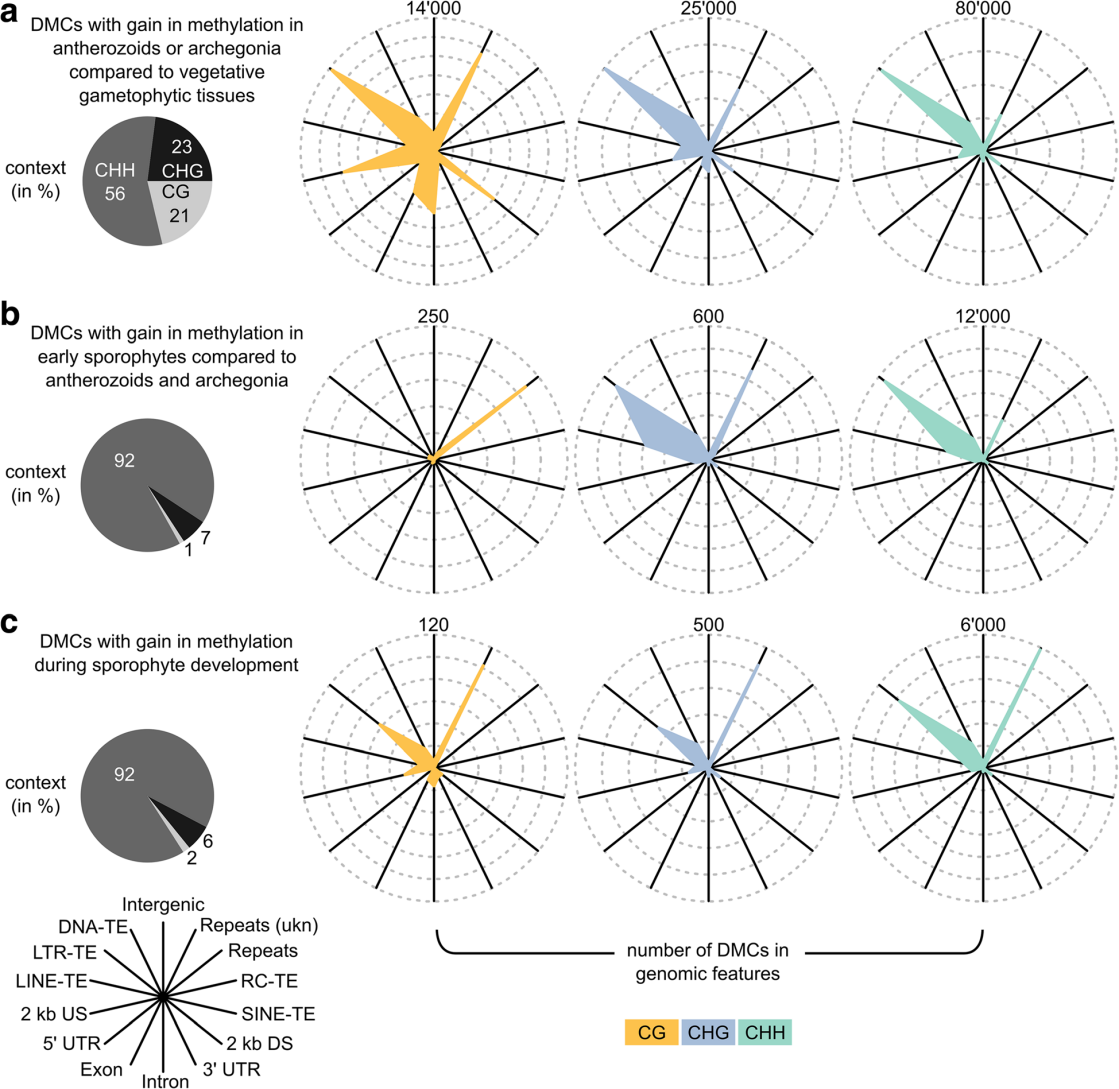
Characterization of DMCs indicative of epigenetic reprogramming, i.e. DMCs that gained methylation in (**a**) antherozoids or archegonia compared to vegetative gametophytic tissues (309,834 DMCs), (**b**) early sporophytes compared to gametangia/gametes (24,073 DMCs), and (**c**) during sporophyte development (15,549 DMCs). Their distribution across the three sequence contexts (CG, CHG, and CHH) is shown as *pie charts* on the *left*. For each sequence context, the number of DMCs associated with a given genomic feature context is shown in a *spider graph* on the *right*. TE transposable element, LTR long terminal repeat, LINE long interspersed element, SINE short interspersed element, RC rolling circle transposon, US/DS upstream/downstream gene flanking regions, UTR untranslated region. “Repeats” included satellite, telomeric satellite, rRNA, snRNA, and simple repeats. “Repeats (ukn)” included unknown and unclassified repeats

To investigate the mechanisms underlying these changes in DNA methylation, we characterized the three sets in terms of their sequence context and their distribution across genomic features (Fig. 5). The DMCs in sets (2) and (3) were similar to each other, with most DMCs being in the CHH context (92% in both), and located in LTR retrotransposons, DNA transposons, and unknown repeats (Fig. 5b, c). The main difference was that DMCs in set (2) were more often found in LINE retrotransposons and LTR retrotransposons but less frequently in unknown repeats than the DMCs from set (3) (all *P* < 0.0001, see Additional file 1: Tables S11–S13 for enrichment analyses of all features and contexts). However, given the relatively low number of DMCs in the two sets, it is unclear whether this reflects real biological differences.

The DMCs in set (1) were clearly different from sets (2) and (3). They frequently occurred in all sequence contexts (21%/23%/56% in the CG/CHG/CHH context, respectively) and showed enrichment in the CG and CHG contexts compared to the background (i.e. among 1 million tested Cs, + 5% in the CG, + 9% in the CHG, – 15% in the CHH context, respectively; see Additional file 1: Figure S3A, “tested”). Similar to the DMCs in sets (2) and (3), the DMCs of set (1) in the CHG and CHH context were most often found in LTR retrotransposons, DNA transposons, and unknown repeats (Fig. 5a). However, many were also located in genes (CHG = 11.1%, CHH = 5.9%) and gene flanking regions (2 kb upstream and downstream, CHG = 17.3%, CHH = 15.6%). The enrichment in genes and gene flanking regions was even more pronounced for the DMCs in the CG context (genes = 21.0%, flanking regions = 26.7%). Interestingly, < 20% of the DMCs located in genes were also located in TEs or repeats (i.e. regions where genes and transposons or repeats overlap, CG = 14.0%, CHG = 16.9%, CHH = 22.4%). This trend was less pronounced for the DMCs in gene flanking regions where up to 55% were also located in TEs or repeats (CG = 30.0%, CHG = 41.8%, CHH = 54.7%). Nonetheless, these results clearly suggest that the enrichment of DMCs in genes and gene flanking regions was not solely caused by TEs or repeats interspersed between genes and their flanking regions.

The enrichment in the CG and CHG contexts and the gene bodies and gene flanking regions of the DMCs in set (1) compared to the DMCs in sets (2) and (3) suggests a difference in the mechanism underlying reprogramming in gametangia/gametes compared to the one acting in the sporophyte. To explain these differences, we analyzed the expression of genes involved in DNA methylation and DNA demethylation, RNA-directed DNA methylation (RdDM), and siRNA processing in publicly available datasets (see Additional file 1: Figure S5, data from [25]). Since these data were collected using entire gametophores instead of archegonia and antherozoids, we analyzed the expression of DNA methyltransferases in the same tissues that we used in our analysis of DNA methylation (see Additional file 1: Figure S6).

The expression pattern of all genes involved in DNA methylation in the archegoniophores (the structure carrying the archegonia) closely resembled the ones in gametophytic thalli. In general, the DNA methyltransferases are relatively lowly expressed in these tissues, yet the isolated archegonia exhibited a clear increase in expression of Mp*DRMb* compared to the gametophytic tissues (see Additional file 1: Figure S6). Genes with an increased expression in the antheridiophore encoded the DNA methyltransferases MpMET and MpCMTa, the PIWI-domain containing proteins MpPIWIa and MpPIWIb (siRNA processing), and the RNA methyltransferase MpHEN (RdDM). Mp*DRMa* and Mp*CMTa* were highly expressed in both antheridiophores and antherozoids, whereas Mp*DRMb* showed only high expression in antherozoids, indicating differential regulation. In young sporophytes, all DNA methyltransferases were expressed, characterized by high expression of Mp*DRMb* and a preferential expression Mp*CMTb* (see Additional file 1: Figure S6). Interestingly, mature sporophytes exhibit DNA methyltransferase expression levels similar to gametophytic tissues, even though an increase in DNA methylation in the CHH context is observed at this stage (see Additional file 1: Figure S6).

Thus, the occurrence of DNA methylation in gene bodies and the enrichment of the CG and CHG context in the antherozoids may be explained by an antherozoid-specific RdDM machinery (reflected by the very high expression of Mp*HEN*) and the high expression levels of all the genes encoding the DNA methyltransferases, with MpMET likely enforcing the high levels of DNA methylation in the CG context. Likewise, the elevated levels of DNA methylation in the CHG context might be explained through maintenance by MpCMTa, MpCMTb, and the DRM methyltransferases MpDRMa and MpDRMb. However, this remains speculative because the CMTs in *M. polymorpha* belong to a clade distinct from those in *A. thaliana* [25] and only a few CMTs were experimentally shown to be capable of maintaining DNA methylation. In *A. thaliana*, CMT3 is required for CHG methylation [33, 34] and CMT2 was found to be associated with CHH methylation [35, 36] and de novo methylation in the CHG and CHH contexts [37]. Furthermore, the correlation between loss of both CMT3 and gene body methylation in the crucifers *Eurema salsugineum* and *Conringia planisiliqua* indicates an important role of CMT3 in CG and CHG methylation [38]. CMT3 is specific to angiosperms and does not occur in *M. polymorpha* [39]. However, the closest known homolog of Mp*CMTa* and Mp*CMTb*, the *CMT* gene of *Physcomitrella patens* (*PpCMT*), has recently been shown to be involved in the maintenance of DNA methylation [40]. Interestingly, Mp*CMTa* appeared to be slightly more closely related to *PpCMT* than Mp*CMTb* [25]. Thus, it would be interesting to investigate whether Mp*CMTa* and Mp*CMTb* are functionally different, with Mp*CMTa* being involved in the maintenance of DNA methylation and Mp*CMTb* being involved in de novo DNA methylation, or exhibit a preference for a specific context.

Interestingly, increasing DNA methylation in the CHH context during sporophyte development in *M. polymorpha* resembles the pattern found in *A. thaliana*, in which CHH methylation increases during embryo (i.e. sporophyte) development [16]. This increase in CHH (and CHG) methylation, specifically in TEs, was explained by the activity of CMT2 and the RdDM pathway [16]. Intriguingly, in *M. polymorpha*, Mp*CMTb* seems to be preferentially expressed in young sporophytes (see Additional file 1: Figures S6) and almost all genes of the RdDM pathway and most DNA methyltransferases are expressed as well at a higher level in young sporophytes compared to vegetative gametophytic tissue and old sporophytes (see Additional file 1: Figures S5). It would be fascinating to see whether this mechanism is conserved in plants.

Also unresolved remains the contribution of Mp*ROS1a*, for which the *A. thaliana* homolog ROS1 acts as DNA demethylase with a preference for a non-CG context [41, 42]. It reaches its highest expression in antheridiophores and sporophytes. However, DNA methylation levels in the antherozoids seem largely unaffected by it. Aside the possibility that the high expression observed in antheridiophores is not reflected in the antherozoids, it is also possible that activity of Mp*ROS1a* is delayed in the antherozoids and starts only after fertilization (e.g. by delayed translation). This would therefore provide an explanation for the strong decrease in DNA methylation in sporophytes compared to the gametangia/gametes. However, it remains to be shown that Mp*ROS1a* acts indeed like its *A. thaliana* homolog *ROS1*.

### Genes with gene body methylation are enriched in epigenetic and regulatory functions

To characterize the genes affected by gene body methylation in antherozoids or archegonia compared to vegetative gametophytic tissues, we extracted all genes with at least ten DMCs in their gene body (1183 genes, 44.5% of them with a gene ontology [GO] term) and performed a GO term enrichment analysis with the terms belonging to the domain “biological process” [43]. We could identify 14 terms with significant enrichment in the set of genes affected by gene body methylation (Table 1). Among them were “DNA-templated regulation of transcription” (GO:0006355), “histone modification” (GO:0016570), “histone lysine methylation” (GO:0034968), “gene silencing by RNA” (GO:0031047), “mRNA splice site selection” (GO:0006376), “RNA processing” (GO:0006396), and “chromosome organization” (GO:0051276). Interestingly, these results suggest that de novo gene body DNA methylation in antherozoids or archegonia is biased towards genes involved in epigenetic regulation, transcription, and DNA/RNA metabolism. Expression of these genes was slightly elevated in the antheridiophore carrying the antherozoids compared to other tissues (see Additional file 1: Figure S7, data from [25]). However, whether gene body methylation within these genes has an influence on gene expression or is a by-product of active transcription remains an open question [38, 39, 44].

**Table 1:** List of GO terms found to be significantly enriched in the set of genes with at least ten DMCs reflecting a gain of methylation in their gene bodies in archegonia or antherozoids compared to vegetative gametophytes

Term ID	Term	Annotated	Selected	Expected	*P* value
GO:0007017	Microtubule-based process	86	18	5.17	<0.00001
GO:0006355	Regulation of transcription (DNA-templated)	239	29	14.36	0.00018
GO:0016570	Histone modification	18	7	1.08	0.00291
GO:0071586	CAAX-box protein processing	2	2	0.12	0.00360
GO:0034968	Histone lysine methylation	7	3	0.42	0.00627
GO:0031047	Gene silencing by RNA	7	3	0.42	0.00627
GO:0006376	mRNA splice site selection	3	2	0.18	0.01036
GO:0006075	(1- > 3)-beta-D-glucan biosynthetic process	3	2	0.18	0.01036
GO:0042147	Retrograde transport (endosome to Golgi)	3	2	0.18	0.01036
GO:0006396	RNA processing	146	20	8.77	0.03011
GO:0007275	Multicellular organism development	12	3	0.72	0.03149
GO:0000079	Regulation of cyclin-dependent	5	2	0.30	0.03187
	Protein serine/threonine kinase activity				
GO:0051276	Chromosome organization	65	15	3.91	0.03608
GO:0006558	L-phenylalanine metabolic process	6	2	0.32	0.04593

“Annotated” corresponds to the total number of all genes in *M. polymorpha* annotated with the given GO term (reference set). “Selected” refers to the number of genes with at least ten DMCs annotated with the given GO term (test set). “Expected” gives the number of genes, which would be expected to be annotated with the given GO term if the test set were randomly sampled from the reference set

*mRNA* messenger RNA

## Conclusion

To determine the DNA methylation dynamics during the life cycle of *M. polymorpha*, we isolated several tissues at various developmental stages. We first characterized the DNA methylation patterns of the individual tissue types separately (Fig. 2) and then focused on the identification of DMCs to characterize the extent of DNA methylation dynamics during the *M. polymorpha* life cycle. DNA methylation varied greatly between the different tissue types with 42% of all tested positions being identified as DMCs. However, the differences were clearly limited to a few comparisons and suggested four major epigenetic landscapes specific to the sporophytes, the gametophytes, the antherozoids, and the archegonia (Fig. 4). DNA methylation was generally low in the gametophytes and DNA methylation dynamics during the life cycle were largely driven by a gain of DNA methylation in antherozoids and archegonia, a loss in the (early) sporophyte compared to the antherozoids and the archegonia before fertilization, and another gain during sporophyte development.

Characterization of three sets of DMCs indicative of epigenetic reprogramming, based on the fact that a gain in DNA methylation requires an active process, suggests that distinct mechanisms underlie reprogramming at different stages of *M. polymorpha* development (Fig. 5). DMCs with a gain in methylation in antherozoids and archegonia relative to the gametophytes showed a clear enrichment in the CG and CHG contexts and the gene bodies and gene flanking regions. In contrast, gain of DNA methylation during sporophyte development was mostly found in the CHH context, LTR retrotransposons, DNA transposons, and repeats, which partially resembled the CHH methylation pattern dynamics observed during early *A. thaliana* sporophyte development [16]. Some of the differences could be explained with previously published expression data [25], leading to the speculation that at least one of the CMTs present in *M. polymorpha* might act as a maintenance methyltransferase. However, the exact mechanisms underlying epigenetic reprogramming in the gametes and the sporophyte remain to be elucidated.

We do not know whether resetting of DNA methylation is complete or if certain positions evade erasure. An experiment including more generations and a sequencing depth reaching saturation would be required to answer these questions. Nonetheless, our study clearly demonstrates that there is extensive reprogramming in *M. polymorpha* and that distinct mechanisms are involved in this process in the gametophytic and the sporophytic generation. Finally, our datasets provide valuable information for in-depth studies of DNA methylation at specific genes of interest, further expanding the genomic resources of the emerging model system *M. polymorpha*.

## Methods

### Plant material and growth conditions

Male (Tak-1) and female (Tak-2) *M. polymorpha* strains were originally obtained from T. Kohchi (Kyoto University) and J. Haselhoff (University of Cambridge) in 2012 (described in [45]). The *M. polymorpha* reference genome is based on Tak-1 [25]. To avoid a bias caused by different alignment efficiencies or strain-specific methylation patters, strains were crossed repeatedly to equalize the genomic background. Plants were grown from spores on sterile half-strength Gamborg B5 basal medium (PhytoTechnology Laboratories) in a growth chamber at 22 °C under fluorescent light under long-day conditions (16 h light, 8 h dark) for six weeks. Plants were then transferred to soil (“Einheitserde D73,” Universalerde mixed in a 1:1 ratio with sand). Positions in the growth chamber were randomized twice a week. Sexual reproduction was induced two weeks after transfer to soil by supplementing with far-red light (740 nm, GreenPower LED module HF far-red, #929000464503, Philips). Fertilization was carried out manually. To isolate antherozoids (sperms), a drop of sterile water (around 15 μL) was placed on top of the male gametophore. Sperms were then released into the water in approximately 1 min and the droplet containing the antherozoids was collected. The droplet was deposited at the ventral side of the female receptacle. We used three male and three female individuals for the entire study and each individual was used once in a cross.

### Tissue harvesting

Male and female thalli and apical notches, antherozoids, archegonia, and sporophytes were collected at distinct developmental stages using different isolation methods. Thalli and apical notches were isolated just before induction of sexual reproduction on the same day and at a similar time. Male and female thalli and apical notches were perforated and collected with straight cylindric tubes with diameters of 5 mm and 3 mm, respectively. Three/five pieces were collected from different lobes of one individual for each thallus/apical notch sample. The obtained thallus samples contained some rhizoids attached to the ventral surface, but no gemma cups. Antherozoids were isolated as described above for the manual fertilization procedure. For each sample, we collected and pooled antherozoids from about three antheridiophores (total volume of 45 μL). Archegonia were collected by dissecting the entire archegoniophore, followed by manual isolation of the archegonia under a dissecting scope (LEICA MZ 6, Leica Microsystems AG). For each sample we collected at least 600 archegonia from about 12 different archegoniophores from one individual. Early and late sporophytes were collected two and four weeks after fertilization, respectively. Sporophytes were collected by dissecting the female gametophore, followed my manual isolation of the developing sporophyte. We isolated around ten sporophytes per sample. All tissues were snap-frozen in liquid nitrogen and stored at – 80 °C until further use. Each tissue was collected from all three available individuals or crosses, resulting in a total of 24 samples (see Fig. 1 for an overview).

### Whole-genome bisulfite sequencing

DNA was extracted from frozen plant material using the MasterPure™ DNA purification kit (Epicentre) following the protocol “Plant Leaf DNA Purification Protocols” with a customized tissue-grinding step. To ensure homogenous grinding of each sample, we added five glass beads (2 mm diameter) to the tubes with frozen plant material, snap-froze the tissue again in liquid nitrogen, and ground the tissue three times for 12 s in a Silamat S6 mixer mill (Ivoclar Vivadent). Isolation and purification of DNA was followed by the fragmentation of the samples to an average sequencing library insert size of 350 bp using the Covaris S2 system (Covaris). Sequencing libraries were produced individually per tissue and replicate from the fragmented DNA, using the HTP Library Preparation Kit for Illumina platforms, following the corresponding protocol (Kapa Biosystems). In order to assess the DNA methylation status of the samples, we included an additional bisulfite treatment in the KAPA KTP library preparation protocol. The bisulfite conversion was carry out using the EpiTect Bisulfite Kit (Qiagen) and performed after DNA adapter ligation but before the amplification of the libraries. Samples were individually barcoded using the Illumina-compatible SeqCap adapter Kits A and B (Roche). Quality of the sequencing libraries was assessed on an Agilent 2100 Bioanalyzer instrument (Agilent). Libraries were paired-end sequenced (2 × 125 bp) on the Illumina HiSeq 2500 system (24 libraries on three lanes). Short reads were deposited at SRA (SRP101412, [46]).

### Alignment of WGBS reads

Reads were quality-checked with FastQC (bioinformatics.babraham.ac.uk/projects/fastqc). Following removal of adaptor sequences and low-quality bases with TrimGalore (version 0.4.1 with the parameters --illumina --paired --clip_R2 2, www.bioinformatics.babraham.ac.uk/projects/trim_galore), reads were aligned to the *M. polymorpha* reference genome (v3.1 from Phytozome 11, [25, 47]) using Bismark (version 0.16.3 [48]) in conjunction with Bowtie 2 (version 2.3.0 [49]) with the parameters --bowtie2 --seedmms 1. Clonal reads with identical sequences resulting from possible over-amplification during sample preparation were marked with Picard (version 2.3.0, broadinstitute.github.io/picard). Methylated and unmethylated read counts for all cytosines in the CG, CHG, and CHH context were finally obtained with PileOMeth (github.com/dpryan79/PileOMeth). Cytosines with a coverage < 5 or > 100 were removed to avoid a potential bias originating from low coverage or poorly annotated sequences [31]. The number of cytosines passing this filter and coverage statistics are given in Supplemental Table S1. Bisulfite conversion rates were on average 98.5% as assessed from unmethylated plastid genomes (see Additional file 1: Table S1).

### Gene annotation and identification of TEs and repeats

We used the annotation available from Phytozome (v 3.1, [47]). Given that we could only use the annotation with the genes (the file with the repeats contained identifiers that could not be assigned to repeat or transposon families), we identified TE and repeat regions with RepeatModeler (version 1.0.8 [50]) and RepeatMasker (version 4.0.6 [51]), and merged the resulting annotation with the gene annotation (available online [52]). For Fig. 5 we summarized the TE and repeat classes into a few distinct groups: (1) DNA transposon; (2) LTR transposon; (3) LINE transposon; (4) SINE transposon; (5) RC/Helitron transposon; (6) repeats (comprises satellite, telomeric satellite, simple, rRNA, and snRNA repeats); and (7) unknown repeats (comprises unclassified and unknown repeats). It is noteworthy that we found some transposons belonging to LINE families other than L1 and RTE. To the best of our knowledge, there have been no previous reports of transposons belonging to these LINE families in plants. In the reference genome paper [25], only LINE L1 and RTE are mentioned. We verified our repeat analysis with the *A. thaliana* reference genome (arabidopsis.org) and an assembly of *Boechera divaricarpa* (data not shown). In both cases, we could only identify the LINE-L1 and LINE-RTE groups (LINE-RTE only in *B. divaricarpa*). Thus, it is unlikely that the results from *M. polymorpha* were exclusively false positives.

### Mapping of genomic positions to local genetic context

Genomic positions (e.g. DMCs with a gain in methylation during sporophyte development) were mapped to their local feature context using the annotation containing genes, gene-flanking regions (2 kb upstream and downstream), TEs, and repeats. Regions without annotation were defined as intergenic. Genes were further broken down into exons, introns, 5' UTRs, and 3' UTRs. For methylation statistics (e.g. Fig. 5), annotations were given equal priorities (except for unknown and unclassified repeats, which were given lower priorities) and their score was increased by the fraction of the number of features that mapped to the position.

### Comparison of genome-wide DNA methylation levels

Variation in average genome-wide methylation levels (see Additional file 1: Table S3 and S8) in fractions (percentage/100) was analyzed with a general linear model in R [53], according to crossed factorial designs with the two/three explanatory factors “sequence context,” “tissue type,” and “feature,” and all interactions between them. The factor “feature” was only included in the second analysis (see Additional file 1: S8). The model was fitted with the glm() function using the “quasibinomial” distribution (i.e. binomial with over-dispersion) and the “logit” link. Such a model is similar to a model using the beta-binomial distribution except for the over-dispersion being a constant instead of a linear function of the number of individuals included in the model [54]. The model was then tested for significance with an analysis of deviance [54] (which is the same as an ANOVA but using deviances instead of variances).

### Determination of DMCs across all conditions

For the analysis of differential methylation, we only used cytosines that were sequenced in at least two out of three replicates across all tissue types (1,005,661 in total, 994,696 without X and Y chromosome scaffolds, which we excluded given that they do not occur in all tissue types). Variation in DNA methylation at each individual cytosine was then analyzed with a linear model in R [53] with the package DSS (version 2.24.0; [14]), according to a design with a single factor comprising all different experimental groups (similar to the approach described for RNA-sequencing [55]). Specific groups were compared with linear contrasts and *P* values were adjusted for multiple testing (Benjamini–Hochberg, i.e. FDR). Cytosines with an adjusted *P* value (FDR) < 0.001 were considered to be differentially methylated and termed DMCs. Differences in DNA methylation levels at individual cytosines were calculated as the difference between the average methylation levels between the two groups. If a group contained more than one tissue type (e.g. early sporophyte compared to antherozoids and archegonia), we generally used the average across average methylation levels within this group to calculate the difference in DNA methylation (Fig. 4, see Additional file 1: Figure S4). However, for the analysis of DMCs with a clear gain in DNA methylation (Fig. 5), we used the highest average methylation level within the groups to calculate the difference in DNA methylation and filtered for a minimal gain of 10%. We chose this approach, favoring loss over gain, because we were interested in identifying DMCs that unambiguously gained DNA methylation (Fig. 5, see Additional file 1: Figures S11–S13).

The key advantage of the approach using a single model and all data, compared to multiple separate pairwise comparisons, is that all tests are performed on the same data with the same residual estimates. It thereby grants a more balanced (same positions, same residual estimates) and more powerful (residual estimates are always based on all available samples) analysis. In contrast, multiple pairwise comparisons use different data and residual estimates in each individual comparison. The data used for a given test depends on the comparison, because a given position must have data in all conditions used in the test. If genome coverage is not saturated (like in this study), the size of the dataset used in a comparison will decrease as the number of conditions increases. In parallel, the residual estimates are more reliable with more samples. Thus, in a pairwise comparison with many samples (e.g. gametes vs gametophytes with a total of 18 individuals), there will be few tests with a high statistical power. In contrast, a pairwise comparison with few samples (e.g. antherozoids compared to archegonia using only six individuals) will perform more tests with less statistical power. Considering that corrections for multiple testing depend on the number of tests performed, the differences between the two pairwise comparisons will be further increased. Interpretation of a collection of pairwise comparisons is therefore difficult and probably strongly driven by the imbalance between the comparisons. Thus, even though the approach with a single linear model will only use positions for which data is available in all conditions, the results are more comparable to each other, thereby allowing for a better interpretation of global patterns of differential DNA methylation.

### GO enrichment

To functionally characterize the genes containing DMCs, we tested for enrichment of GO terms with topGO (version 2.26 [43]) in conjunction with the GO annotation available from Phytozome [47]. Analysis was based on gene counts (genes with DMCs compared to all annotated genes) using the “weight” algorithm with Fisher’s exact test (both implemented in topGO). A term was identified as significant if the *P* value was < 0.05.

### Droplet digital polymerase chain reaction (ddPCR) for expression analysis of DNA methyltransferases

For RNA extraction, *M. polymorpha* tissue samples were harvested using the same methods as for DNA extraction. Three biological replicates were harvested from plants cultured simultaneously or at a different time-point but under identical conditions as for DNA extraction. Archegonia (around 400 per replicate) and antherozoid samples (extracted from five or more antheridiophores) were pooled from two or more plants. Total RNA extraction and DNAse treatment was performed using the Direct(-zol)^TM^ RNA MiniPrep (ZymoResearch) with TRIzol^TM^ Reagent (Ambion), according to manufacturer’s protocol. RNA concentrations were measured using the Qubit RNA HS Assay Kit (Invitrogen) and a Qubit 3.0 Fluorometer (Invitrogen). All antherozoid samples were measured with the 4200 TapeStation system (Agilent), as their corresponding RNA concentrations were below the limit of detection of the Qubit fluorometer. Sample concentration measurements and total RNA input material used in downstream reactions are listed in Additional file 1: Table S14. After RNA extraction and measurement, all samples were converted to cDNA using 200 units of SuperScript® II Reverse Transcriptase (Invitrogen) according to the manufacturer’s protocol in a 25-uL reaction, using 20-μg/mL Oligo(dT)12-18 primers. All samples were controlled in parallel for genomic DNA contamination with RT(–) reactions, in which SuperScript® II Reverse Transcriptase was replaced with nuclease-free water.

Two genes, Mp*APT3* and Mp*ACT7*, reported to be evenly expressed across different *M. polymorpha* developmental stages [56], were used as reference genes. All primers to amplify DNA methyltransferase genes were designed using the CLC Main Workbench (Qiagen) software. The primer sequences used for the analysis are listed in Additional file 1: Table S15. All primers were tested and validated for optimal concentration; primer efficiency was assessed for the five newly designed primers (see Additional file 1: Table S16). Primers were tested in a 7500 Applied Biosystem Fast quantitative Real-Time PCR System and later validated on a QX200 Droplet Digital PCR System (Bio-Rad) for the ddPCR assay. Reactions for real-time PCR were performed in total volumes of 20 μL containing 10 μL 2X SYBR-green Supermix (SsoAdvanced™ Universal SYBR®). For the ddPCR analysis, individual PCR reactions were performed in a total volume of 25 μL, using 1× ddPCR EvaGreen Supermix, with droplets generated according to manufacturer’s recommendations. Reading of the PCR-amplified droplets was carried out by the QX200 Droplet Reader (Bio-Rad) and analyzed by the QuantaSoft™ Software (v1.4, Bio-Rad).

Raw data are provided in Additional file 1: Table S16. To compare the expression of the studied genes between the different tissues, we calculated log2 ratios between the test genes and the reference genes. To this aim, RT(+) counts of the test genes and the reference genes were first log2(x + 1) transformed and the value of the reference genes was then subtracted from the value of the test gene (resulting in two datasets, one with Mp*APT3* and the other with Mp*ACT7* as reference). Tissues were compared with two-sided t-tests. For each gene, *P* values were adjusted for multiple testing. Adjusted *P* values (FDR) < 0.05 were considered to be significant. Even though the primer pair of the Mp*ACT7* reference gene did not span an intron (thus potentially amplifying genomic DNA if present), results were similar to the ones obtained with Mp*APT3* as reference gene (see Additional file 1: Figure S6).

## Additional file

Additional file 1: Supplemental figures and tables. This PDF file contains all supplemental figures and tables. (PDF 7269 kb)

## Abbreviations

DMC: Differentially methylated cytosine
FDR: False discovery rate
LTR: Long terminal repeat
LINE: Long interspersed elements
RC: Rolling circle transposon
RdDM: RNA-directed DNA methylation
SINE: Short interspersed elements
TE: Transposable element, transposon
