# Supplementary material for: Extensive epigenetic reprogramming during the life cycle of Marchantia polymorpha
Source: Genome Biol. 2018 Jan 25;19:9. doi: 10.1186/s13059-017-1383-z (PMC5784723; doi:10.1186/s13059-017-1383-z)
Supplement: Additional file 1: — Supplemental figures and tables. This PDF file contains all supplemental figures and tables. (PDF 7269 kb) [file 13059_2017_1383_MOESM1_ESM.pdf]

Supplemental figures and tables: Extensive epigenetic reprogramming during the life cycle of *Marchantia polymorpha*.

Contents

Supplemental Figures **2**

Supplemental Tables **10**

References **32**

Supplemental Figures

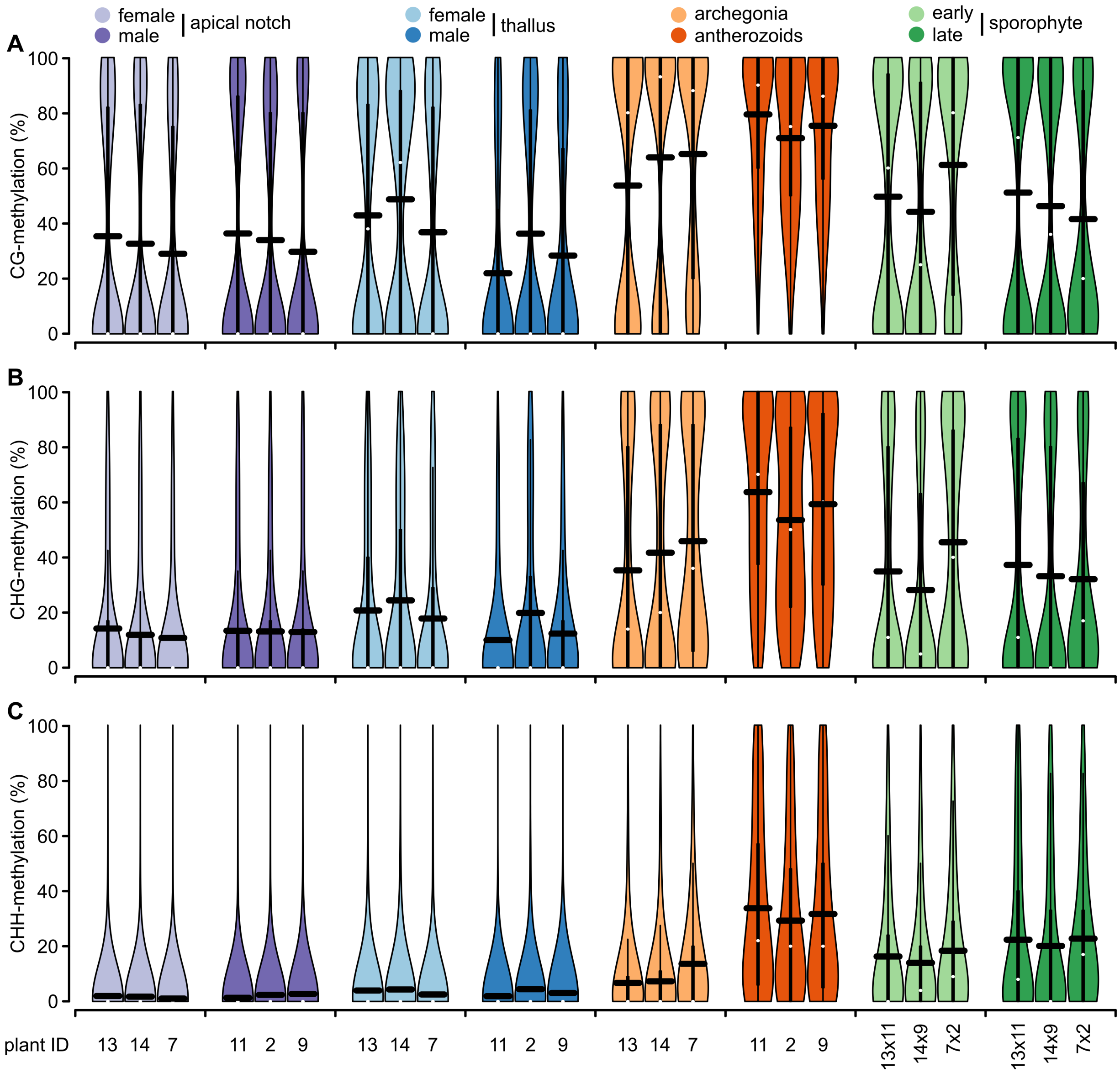

Fig. S1. DNA methylation levels in percent at individual cytosines in the CG (A), CHG (B), and CHH (C) sequence context in all samples used in this study shown as violin plots. Plant IDs for the sporophytic samples indicate both parents (maternal x paternal). The horizontal black bars correspond to the means. For the number of cytosines used for each individual, see Table S1, column “numPF”.

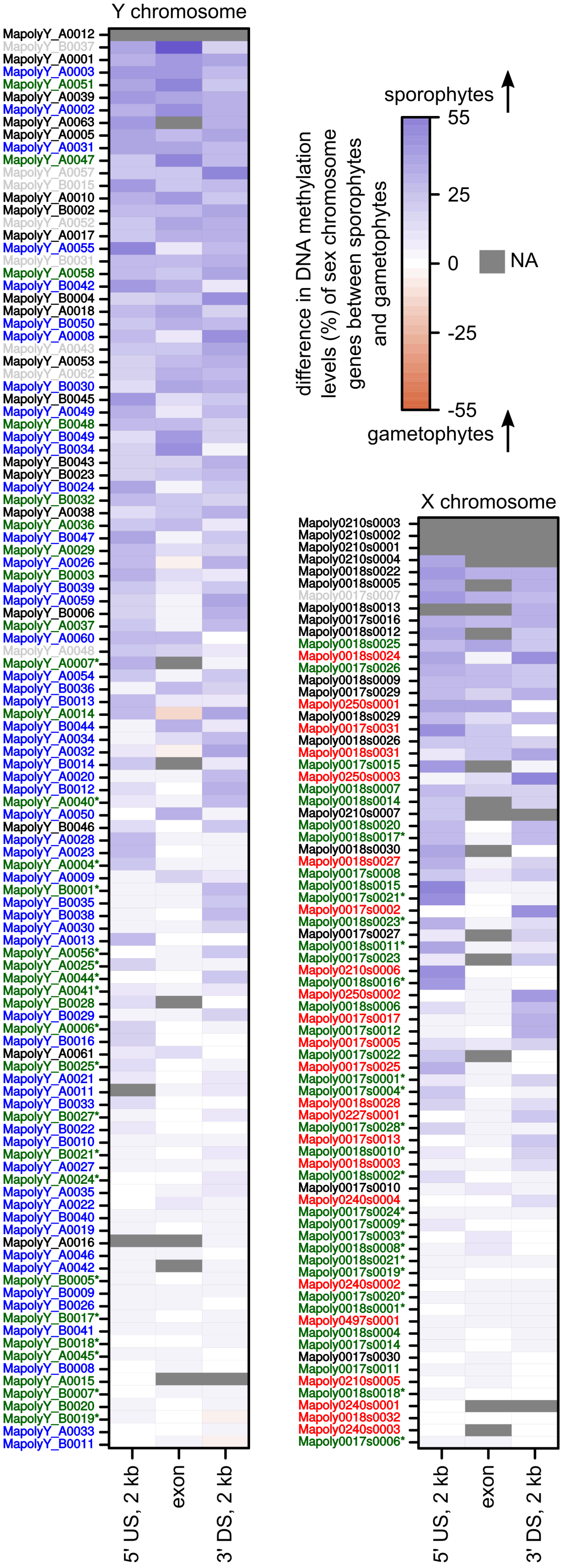

Fig. S2. Difference in overall methylation levels (all contexts) of genes located on the sex chromosomes

(i.e., exons) and their 2 kb flanking regions between sporophytes and gametophytes (US: upstream, DS: downstream). Values in the heatmap correspond to the average of sporophytic tissues minus average of gametophytic tissues (percent methylation difference). Blue/red corresponds to an increased methylation in sporophytes/gametophytes respectively. Gene-IDs are colored according to Figure 2 from [1]. Green: expressed in vegetative and reproductive tissues [1], red/blue: more than ten times higher expressed in reproductive compared to vegetative tissues [1], black: expression not detected [1], gray: not included in the original figure [1]. Genes with an asterisk are shared between the X and Y chromosome [1].

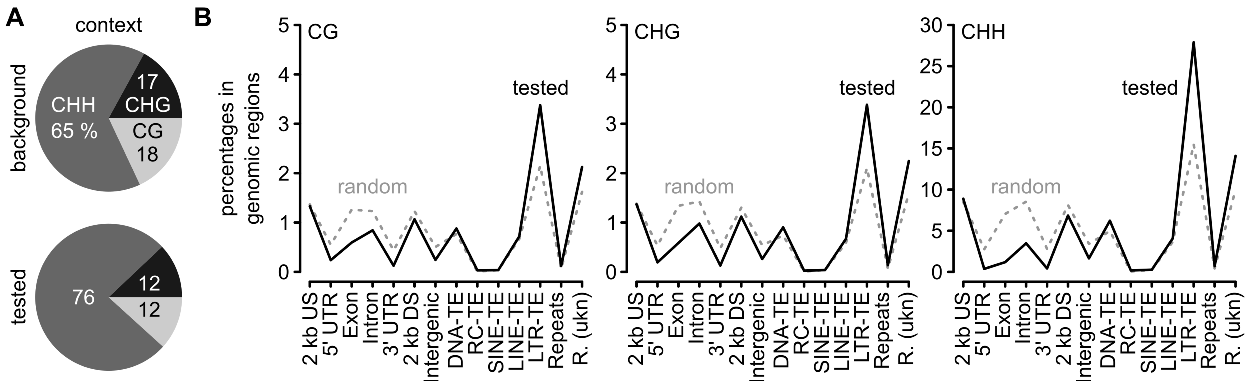

Figure S3. A) Comparison of the sequence context distribution of cytosines used in the analysis of differential methylation (“tested”) with all cytosines in the *M. polymorpha* genome (“background”). B) Comparison of the distribution of the “tested cytosines” across the different genomic features with the distribution obtained from 100 sets of cytosines with identical context frequencies randomly sampled from all cytosines in the *M. polymorpha* genome (“random”). Given the large number of positions (1’005’661 including the sex chromosomes), the random sets were highly similar to each other and only the average is shown (i.e., the tested cytosines are significantly different from the background, $P < 0.01$).

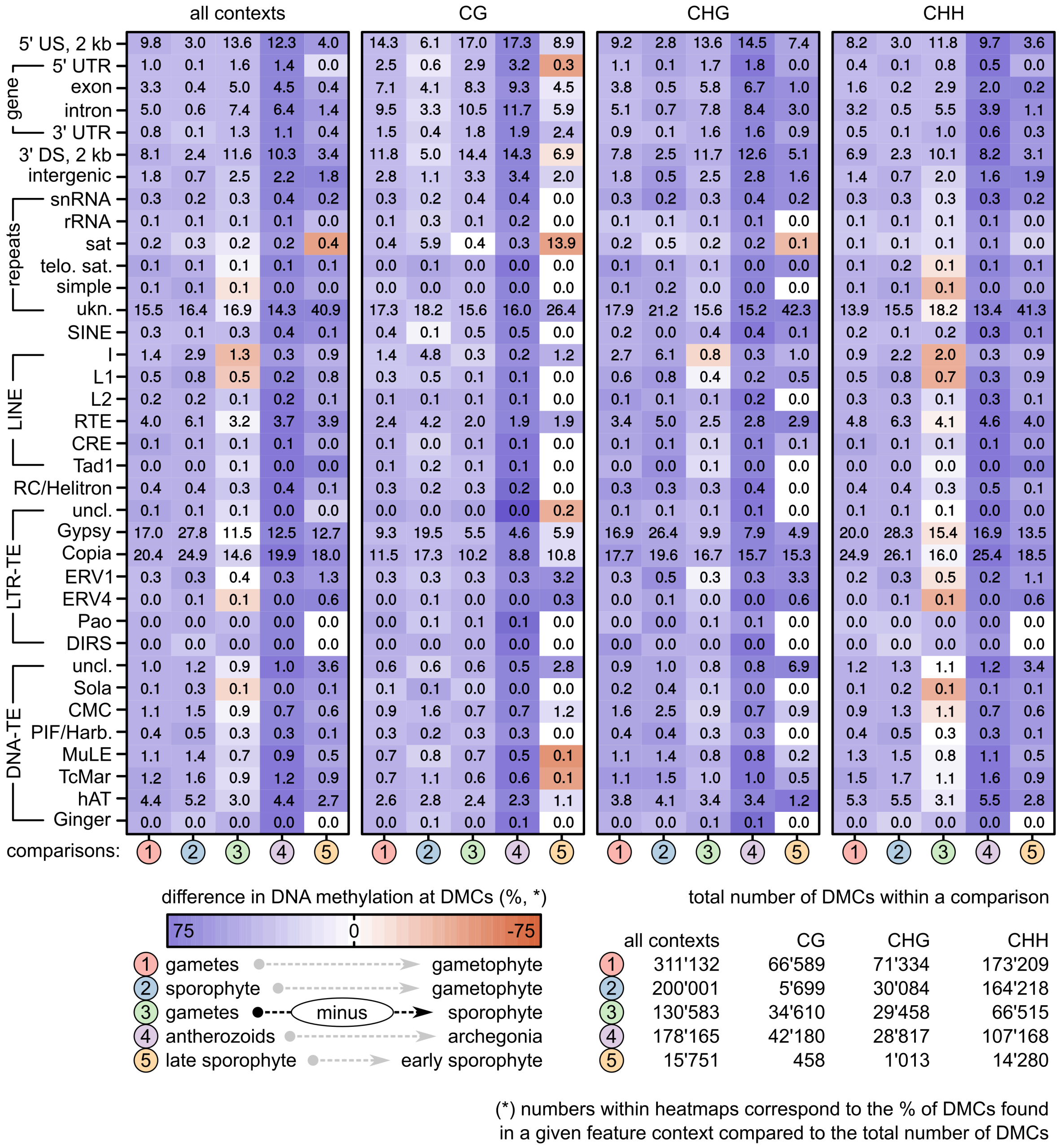

Figure S4. Average differences in DNA methylation at significantly differentially methylated cytosines (DMCs; FDR <= 0.001) within a given sequence and feature context are shown for the five comparisons with a large number of DMCs (gametes *versus* gametophyte, sporophyte *versus* gametophyte, gametes *versus* sporophyte, antherozoids *versus* archegonia, and late sporophyte *versus* early sporophyte). The average differences are shown as color gradient. The numbers within the heatmap refer to the percent of DMCs found within the given feature context compared to the total number of DMCs identified in the comparison. For example, 20.4% of all DMCs identified in comparison 1 (gametes *versus* gametophyte) are located in Copia LTR-TEs and are on average more methylated in the gametes than in the gametophytes. Abbreviations: US/DS upstream/downstream of a gene, UTR: untranslated region, snRNA: small nucleolar RNA, rRNA: ribosomal RNA, sat: satellite repeat, telo. sat: telomeric satellite repeats, simple: simple repeats, ukn.: unknown/unclassified repeats, SINE: short interspersed nuclear elements, LINE: long interspersed nuclear elements, RC: rolling circle, LTR-TE: retrotransposon with long terminal repeats, DNA-TE: DNA transposon, uncl.: not classified into a subfamily.

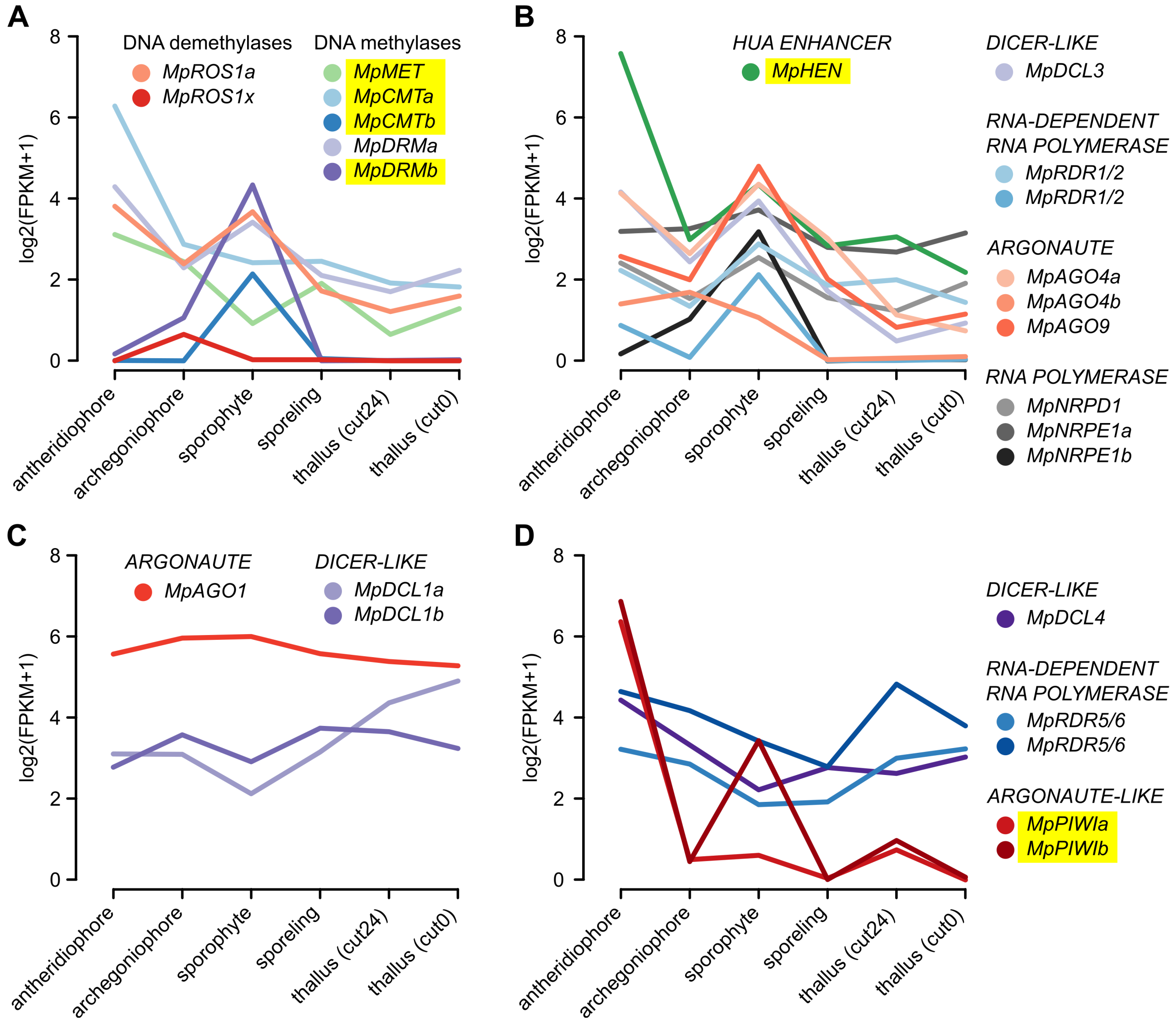

Fig. S5. Expression pattern of DNA methyltransferases and DNA demethylases (A), genes involved in RdDM (B), genes involved in miRNA processing (C), and genes involved in other small RNA processing (D) in *M. polymorpha*. Genes with clear differences in expression between the sporophyte and the antheridiophores were marked in yellow. Data was taken from [1]. Note that the antheridiophore and the archegoniophore are the structures carrying the antherozoids and the archegonia, respectively.

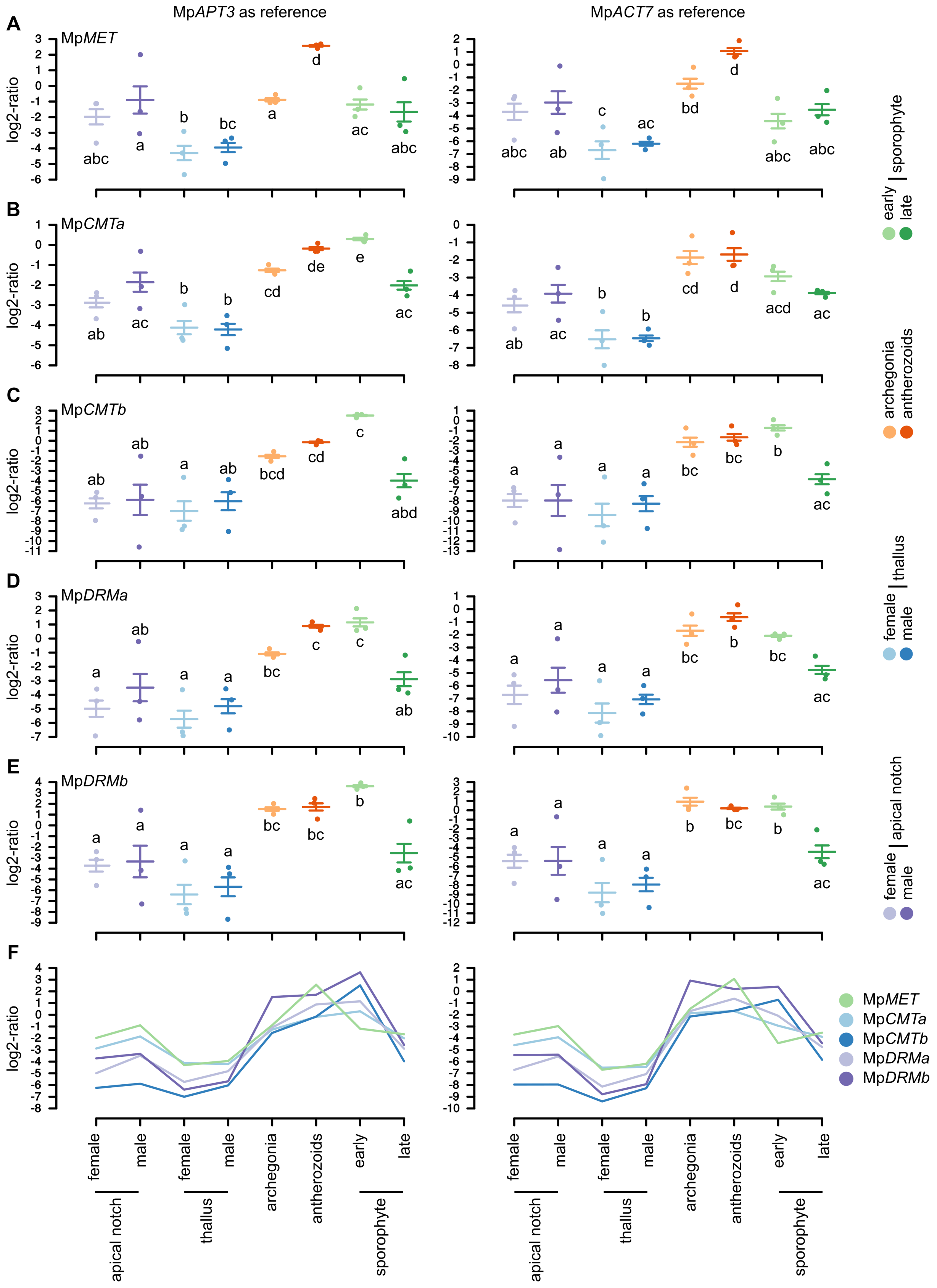

MpAPT3 as reference
MpACT7 as reference
A
MpMET
B
MpCMTa
C
MpCMTb
D
MpDRMa
E
MpDRMb
F
log2-ratio
sporophyte
early
late
archegonia
antherozoids
thallus
female
male
apical notch
MpMET
MpCMTa
MpCMTb
MpDRMa
MpDRMb
female
male
female
male
archegonia
antherozoids
early
late
apical notch
thallus
sporophyte

Figure S6. Expression pattern of DNA methyltransferases in the tissues studies. Expression patterns were assessed with Droplet Digital PCR (ddPCR) with two reference genes for each DNA methyltransferase (Mp*APT3* and Mp*ACT7* in panels on the left and right, respectively). The y-axis corresponds to the log ratio between the test and the reference gene (log2(test gene count + 1) - log2(reference gene count + 1)). A) Mp*MET*, B) Mp*CMTa*, C) Mp*CMTb*, D) Mp*DRMa*, E) Mp*DRMb*, and F) with averages for all genes. A-E) Long horizontal lines indicate the mean, short horizontal lines delimit plus/minus one standard error of the mean. Different letters indicate conditions that are significantly different from each other (two-sided t-test, adjusted for multiple testing, FDR < 0.05). Conditions with the same letter are not significantly different from each other.

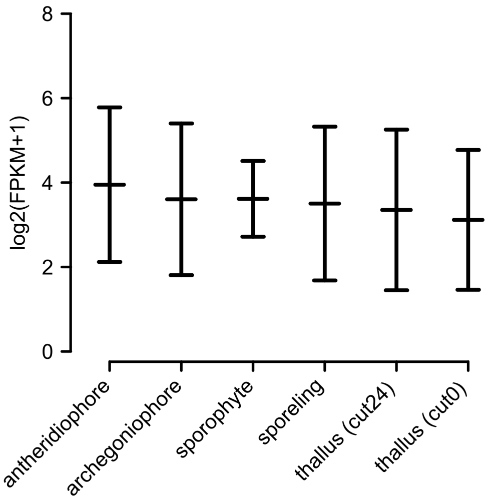

Fig. S7. Average expression (plus/minus one standard deviation) of genes with gene body methylation in antherozoids or archegonia annotated with the GO terms “GO:0090304”, “GO:0031047”, “GO:0006418”, “GO:0006376”, “GO:0000398”, “GO:0006355”, “GO:0016570”, or “GO:0034968” ($n = 38$). Data was taken from [1]. Note that the antheridiophore and the archegoniophore are the structures carrying the antherozoids and the archegonia, respectively.

Supplemental Tables

Table S1. Number of sequenced cytosines and coverage statistics. "totalC" for the total number of cytosines with a coverage greater than zero. "numPF" and "percPF" for the number and percentage of cytosines, respectively, with a coverage of at least 5 and at maximum 100. "totalCov", "meCov", and "unCov" for the total average coverage, the average methylated coverage, and the average unmethylated coverage, respectively (at the cytosines passing the filter). Abbreviations: AN: apical notch, TH: thallus, SPHy: young (early) sporophyte, SPHo: old (late) sporophyte, ANTH: antherozoids, ARCH: archegonia. Individuals number 2, 9, and 11 were male. Individuals number 7, 13, and 14 were female.

sample	chromosomes	totalC	numPF	percPF	totalCov	meCov	unCov
AN_11	autosomal	50950059	11811371	23.18	7.81	0.52	7.3
AN_13	autosomal	50818414	8670594	17.06	7.36	0.61	6.75
AN_14	autosomal	62041600	19446423	31.34	8.04	0.58	7.46
AN_2	autosomal	56190382	17001218	30.26	8.99	0.67	8.32
AN_7	autosomal	46352244	5988758	12.92	6.61	0.38	6.23
AN_9	autosomal	62357490	14928398	23.94	6.86	0.55	6.31
ANTH_11	autosomal	64394498	21533714	33.44	8.16	3.47	4.69
ANTH_2	autosomal	72223764	40203720	55.67	10.23	3.74	6.49
ANTH_9	autosomal	68305752	31868426	46.66	9.7	3.95	5.75
ARCH_13	autosomal	34936725	3018331	8.64	6.84	1.12	5.72
ARCH_14	autosomal	37838484	4813969	12.72	7.57	1.5	6.06
ARCH_7	autosomal	41431921	6030287	14.55	7.52	1.71	5.82
SPHo_13	autosomal	59291188	18180239	30.66	8.11	2.18	5.93
SPHo_14	autosomal	59535590	18453822	31	7.9	1.89	6
SPHo_7	autosomal	66039559	20083165	30.41	7.29	1.82	5.47
SPHy_13	autosomal	57156024	18172698	31.79	8.69	1.86	6.83
SPHy_14	autosomal	68020457	35244138	51.81	12.32	2.36	9.97
SPHy_7	autosomal	40562953	6564298	16.18	7.52	1.89	5.63
TH_11	autosomal	45891319	4139360	9.02	6.38	0.32	6.07
TH_13	autosomal	44433471	4785888	10.77	7.03	0.82	6.21
TH_14	autosomal	36923186	2540033	6.88	7	1.03	5.97
TH_2	autosomal	48686771	10878091	22.34	8.84	0.76	8.08
TH_7	autosomal	36249142	5164197	14.25	7.76	0.57	7.19
TH_9	autosomal	26868081	1097297	4.08	7.07	0.47	6.6
AN_11	plastids	105291	37083	35.22	44.12	0.1	44.03
AN_13	plastids	104916	43405	41.37	42.49	0.18	42.31
AN_14	plastids	106628	26262	24.63	47.22	0.2	47.02
AN_2	plastids	104688	40041	38.25	43.77	0.37	43.39
AN_7	plastids	103696	46532	44.87	40.67	0.07	40.6
AN_9	plastids	105229	28035	26.64	45.92	0.53	45.39
ANTH_11	plastids	39068	18291	46.82	27.91	0.68	27.23
ANTH_2	plastids	46196	20111	43.53	31.27	0.66	30.62
ANTH_9	plastids	46609	17645	37.86	33.34	0.79	32.55
ARCH_13	plastids	103786	57146	55.06	38.8	0.61	38.18
ARCH_14	plastids	104065	61997	59.58	37.86	0.6	37.26
ARCH_7	plastids	102888	62244	60.5	36.25	0.59	35.65
SPHo_13	plastids	104065	36319	34.9	44.71	0.48	44.23
SPHo_14	plastids	105075	22031	20.97	48.18	0.57	47.62
SPHo_7	plastids	104726	21075	20.12	47.08	3.99	43.09
SPHy_13	plastids	103605	46234	44.63	42.03	0.42	41.61
SPHy_14	plastids	104393	34748	33.29	45.2	0.85	44.35
SPHy_7	plastids	102633	64431	62.78	32.52	0.63	31.89

sample	chromosomes	totalC	numPF	percPF	totalCov	meCov	unCov
TH_11	plastids	102270	47763	46.7	41.23	0.09	41.14
TH_13	plastids	103428	51708	49.99	42.4	0.44	41.96
TH_14	plastids	101473	61604	60.71	36.16	0.33	35.82
TH_2	plastids	99393	61164	61.54	34.15	0.3	33.85
TH_7	plastids	102023	55356	54.26	36.63	0.08	36.55
TH_9	plastids	102597	62377	60.8	34.56	0.49	34.07

Tab. S2. Average DNA methylation levels in percent within a given sequence context and tissue type (as shown in Figure 2A, only autosomes).

	CG	CHG	CHH	average across contexts
female apical notch	32.30	12.32	1.60	15.41
male apical notch	33.31	13.17	2.16	16.21
female thallus	42.76	21.00	3.62	22.46
male thallus	28.88	14.15	3.16	15.40
archegonia	60.86	40.91	9.22	37.00
antherozoids	75.17	58.74	31.55	55.16
early sporophyte	51.63	36.15	16.21	34.66
late sporophyte	46.28	34.14	21.73	34.05
average across tissues	46.40	28.82	11.16	

Tab. S3. Results of an analysis of deviance (i.e., ANOVA with deviances instead of variances) for the average DNA methylation in dependence of the sequence context, tissue type, and their interaction. Abbreviations: Df: degrees of freedom, DVC: deviance change, MDVC: mean deviance change, F: variance ratio, P: probability of type-I error, percDVC: percent of deviance change.

source of variation	Df	DVC	MDVC	F	P	percDVC
sequence context	2	7.68	3.84	383.2	3.1e-30	49.03
tissue type	7	6.77	0.97	96.55	4.5e-26	43.24
interaction	14	0.7304	0.0522	5.21	7.9e-06	4.66
residuals	48	0.4798	0.01			3.06

Tab. S4. Average DNA methylation levels (%) within a given genomic feature and tissue type across all sequence contexts (as shown in Figure 2B, only autosomes). Abbreviations: arch: archegonia, anth: antherozoids, sporo: sporophyte, US/DS: upstream/downstream.

genomic feature	female apical notch	male apical notch	female thallus	male thallus	arch	anth	early sporo.	late sporo.	average across tissues
5' US, 2kb	2.19	2.68	3.43	2.77	7.78	33.29	9.00	10.26	8.93
5' UTR	0.90	1.23	1.77	2.17	5.74	35.02	4.15	4.33	6.91
exon	1.37	1.91	2.60	2.36	8.43	35.51	7.83	7.68	8.46
intron	0.82	1.27	1.45	1.57	5.28	29.99	4.19	5.06	6.20
3' UTR	0.75	1.10	1.41	1.43	5.48	35.29	3.54	4.11	6.64
3' DS, 2kb	2.16	2.61	3.45	2.69	8.27	35.07	8.99	10.23	9.19
intergenic	1.64	2.07	2.08	1.65	5.22	30.21	6.16	8.21	7.15
snRNA	2.76	3.24	4.17	3.34	9.01	33.96	11.81	14.67	10.37
rRNA	1.62	1.94	2.18	2.02	6.14	28.26	7.93	9.18	7.41
satellite	1.64	2.28	1.90	1.67	4.43	18.56	11.61	10.38	6.56
satellite_telo.	18.82	20.97	23.71	16.34	36.12	54.82	42.50	50.53	32.98
simple_repeat	13.87	14.82	18.06	15.97	30.57	48.96	37.91	44.17	28.04
uknRepeats	7.96	8.76	12.18	7.62	20.36	40.52	23.76	27.54	18.59
SINE	1.13	1.64	1.85	1.82	5.31	32.17	6.84	8.09	7.36
LINE_I	32.46	33.36	37.55	29.96	52.97	57.04	56.52	62.43	45.29
LINE_L1	19.23	20.65	23.85	17.61	36.84	54.95	42.22	48.17	32.94
LINE_L2	5.70	6.79	6.75	6.68	15.04	47.60	22.38	29.53	17.56
LINE_RTE	7.83	8.75	10.98	8.54	19.79	42.53	28.40	35.64	20.31
LINE_CRE	10.31	11.74	13.92	11.46	22.99	51.38	32.73	38.83	24.17
LINE_Tad1	1.67	1.95	2.70	2.21	6.83	31.68	7.77	9.15	8.00
RC_Helitron	7.78	8.55	9.48	8.80	18.13	46.65	25.60	31.58	19.57
LTR	16.17	17.78	21.73	16.58	32.02	51.43	38.98	46.01	30.09
LTR_Gypsy	14.30	15.65	19.54	14.29	29.19	49.01	36.21	44.64	27.85
LTR_Copia	8.73	10.05	11.45	8.41	19.58	45.05	27.74	35.72	20.84
LTR_ERV1	4.65	5.24	6.54	4.85	13.80	34.84	17.98	25.80	14.21
LTR_ERV4	2.96	3.00	4.49	2.96	10.15	30.55	11.86	16.78	10.34
LTR_Pao	2.84	3.03	4.58	2.77	9.49	35.76	10.62	10.95	10.00
LTR_DIRS	1.15	1.52	1.85	1.74	5.39	34.78	6.46	14.55	8.43
DNA	9.11	10.42	11.73	9.80	21.17	48.31	29.39	37.18	22.14
DNA_Sola	23.43	24.67	28.93	25.94	43.27	55.78	47.49	54.65	38.02
DNA_CMC	22.46	24.29	28.95	17.44	42.78	53.20	45.53	50.93	35.70
DNA_PIF/ Harbinger	11.52	12.31	14.91	12.79	26.80	48.39	34.82	40.93	25.31
DNA_MuLE	10.98	12.03	14.50	11.77	24.98	48.58	33.08	38.90	24.35
DNA_TcMar	9.62	10.98	12.28	9.65	21.30	49.53	30.62	37.71	22.71
DNA_hAT	10.23	11.75	13.01	10.21	22.88	50.44	31.87	38.24	23.58
DNA_Ginger	1.68	2.26	2.46	1.90	5.49	26.63	11.12	11.82	7.92

Tab. S5. Average CG-DNA methylation levels (%) within a given genomic feature and tissue type (as shown in Figure 2B, only autosomes). Abbreviations: arch: archegonia, anth: antherozoids, sporo: sporophyte, US/DS: upstream/downstream.

genomic feature	female apical notch	male apical notch	female thallus	male thallus	arch	anth	early sporo.	late sporo.	average across tissues
5' US, 2kb	8.88	9.86	12.36	9.65	21.87	59.04	19.59	15.28	19.57
5' UTR	1.57	1.94	2.96	3.89	8.45	50.17	6.38	4.53	9.99
exon	3.29	4.27	5.52	4.65	14.38	54.00	13.23	9.87	13.65
intron	2.09	3.03	3.42	3.38	10.13	52.89	8.36	6.47	11.22
3' UTR	1.70	2.07	3.10	2.74	9.22	53.65	7.17	4.91	10.57
3' DS, 2kb	9.15	9.92	13.03	9.41	24.04	60.25	20.58	16.01	20.30
intergenic	6.18	6.89	5.93	3.77	11.92	58.25	13.70	13.52	15.02
snRNA	13.79	14.46	18.71	15.16	30.21	64.23	27.44	23.74	25.97
rRNA	7.36	8.08	9.26	7.30	16.64	59.33	20.45	14.65	17.88
satellite	5.46	7.19	5.43	4.24	10.60	37.37	35.40	18.49	15.52
satellite_telo.	75.91	79.13	77.20	66.78	90.52	94.17	83.88	85.45	81.63
simple_repeat	58.44	59.74	61.80	53.64	79.37	85.22	72.19	72.67	67.88
uknRepeats	33.95	36.02	43.64	31.37	61.19	75.09	52.77	47.68	47.71
SINE	4.49	5.37	6.39	5.70	12.96	56.75	13.47	10.33	14.43
LINE_I	81.02	83.23	80.82	74.42	93.90	94.68	90.09	90.62	86.10
LINE_L1	70.01	72.66	72.45	60.66	86.48	91.75	79.89	80.41	76.79
LINE_L2	30.76	35.17	30.59	32.69	51.77	78.45	47.30	47.62	44.29
LINE_RTE	45.55	47.59	51.11	42.90	67.77	81.48	62.21	61.03	57.46
LINE_CRE	49.41	54.03	55.20	48.24	65.90	85.71	65.74	66.61	61.35
LINE_Tad1	7.33	7.14	10.92	9.80	19.47	56.02	17.88	13.58	17.77
RC_Helitron	41.88	42.83	45.07	40.66	60.13	81.97	54.66	54.51	52.71
LTR	65.47	70.21	69.66	58.85	83.52	90.68	77.42	78.35	74.27
LTR_Gypsy	67.27	69.40	69.86	59.80	84.61	90.55	78.34	79.58	74.93
LTR_Copia	52.76	55.64	56.19	46.48	72.44	85.54	66.34	67.35	62.84
LTR_ERV1	22.69	24.94	29.00	19.88	44.44	67.15	37.91	40.06	35.76
LTR_ERV4	15.03	13.99	21.93	13.68	36.80	62.96	29.93	26.38	27.59
LTR_Pao	12.36	12.36	17.14	9.91	25.42	76.17	25.58	19.62	24.82
LTR_DIRS	4.88	4.99	6.74	5.06	19.12	66.39	12.64	14.79	16.83
DNA	48.92	51.57	53.44	47.38	69.05	83.23	62.64	62.64	59.86
DNA_Sola	77.96	80.63	77.67	74.35	91.90	94.32	86.08	87.81	83.84
DNA_CMC	70.58	73.97	74.34	57.61	89.05	92.08	83.24	83.28	78.02
DNA_PIF/ Harbinger	50.33	51.98	54.52	46.83	73.33	81.02	66.04	63.98	61.00
DNA_MuLE	50.43	52.79	56.24	46.52	71.34	83.34	66.27	62.93	61.23
DNA_TcMar	52.93	55.45	57.16	48.99	71.87	86.28	66.46	66.29	63.18
DNA_hAT	50.82	54.05	55.06	47.32	71.19	85.15	66.84	65.57	62.00
DNA_Ginger	8.68	11.05	11.74	9.46	17.62	68.09	30.94	22.47	22.51

Tab. S6. Average CHG-DNA methylation levels within a given genomic feature and tissue type (as shown in Figure 2B, only autosomes). Abbreviations: arch: archegonia, anth: antherozoids, sporo: sporophyte, US/DS: upstream/downstream.

genomic feature	female apical notch	male apical notch	female thallus	male thallus	arch	anth	early sporo.	late sporo.	average across tissues
5' US, 2kb	3.08	3.66	5.47	4.45	14.20	46.56	12.54	11.94	12.74
5' UTR	0.94	1.29	1.88	2.23	6.67	40.90	4.03	4.48	7.80
exon	1.57	2.18	2.97	2.69	10.36	41.42	8.29	7.96	9.68
intron	0.92	1.38	1.72	1.85	6.79	37.30	4.46	5.27	7.46
3' UTR	0.76	1.10	1.52	1.42	6.85	44.97	3.55	4.20	8.05
3' DS, 2kb	2.64	3.13	4.87	3.79	14.17	48.19	12.05	11.62	12.56
intergenic	2.38	2.88	3.23	2.32	8.30	42.32	8.04	9.60	9.88
snRNA	3.89	4.38	6.96	5.54	17.73	48.94	17.21	17.92	15.32
rRNA	1.89	1.88	2.70	2.50	10.06	32.67	9.27	9.85	8.85
satellite	1.61	2.20	2.12	1.88	5.35	20.61	10.31	10.97	6.88
satellite_telo.	33.85	37.16	43.51	35.23	69.79	84.52	68.38	71.54	55.50
simple_repeat	27.31	29.93	37.19	34.86	64.88	76.60	64.93	64.74	50.06
uknRepeats	12.27	13.45	20.47	14.40	40.40	58.44	37.31	35.76	29.06
SINE	1.11	1.58	2.05	2.08	7.36	44.89	6.95	7.40	9.18
LINE_I	37.54	39.80	45.54	42.29	71.52	77.22	74.71	77.69	58.29
LINE_L1	37.56	40.05	46.42	37.25	72.36	82.63	68.07	69.13	56.68
LINE_L2	9.38	10.94	12.77	13.77	32.15	65.99	34.25	36.31	26.94
LINE_RTE	15.43	16.58	23.70	18.87	47.46	67.35	48.17	49.97	35.94
LINE_CRE	17.79	18.99	27.73	22.50	48.88	72.42	50.98	52.71	39.00
LINE_Tad1	1.38	1.81	2.81	2.00	9.72	42.78	9.73	10.23	10.06
RC_Helitron	11.55	12.95	15.90	15.13	38.44	70.54	41.22	43.33	31.13
LTR	24.67	30.14	34.36	32.40	59.65	79.08	62.48	65.21	48.50
LTR_Gypsy	30.05	32.41	39.59	32.40	64.15	78.53	63.89	66.55	50.95
LTR_Copia	18.90	20.77	26.23	20.50	49.78	72.58	50.30	53.80	39.11
LTR_ERV1	4.06	4.46	6.77	5.70	21.40	46.11	24.96	30.76	18.03
LTR_ERV4	2.72	2.83	5.42	3.54	18.26	40.04	15.22	16.69	13.09
LTR_Pao	2.34	2.41	5.26	2.32	13.95	52.87	15.73	13.99	13.61
LTR_DIRS	0.68	1.12	2.16	1.37	8.15	50.06	8.26	19.09	11.36
DNA	15.93	18.05	21.97	19.11	45.39	68.86	47.37	50.00	35.83
DNA_Sola	42.11	44.44	49.96	47.99	77.37	85.80	74.71	76.30	62.34
DNA_CMC	30.27	33.60	40.26	30.43	66.46	75.90	66.20	67.84	51.37
DNA_PIF/ Harbinger	22.14	23.22	29.38	25.89	57.89	68.97	55.49	54.51	42.19
DNA_MuLE	21.98	23.32	30.50	26.47	56.26	73.79	55.85	55.13	42.91
DNA_TcMar	17.69	19.83	25.28	21.29	50.30	76.10	52.85	55.53	39.86
DNA_hAT	19.35	21.71	26.52	22.08	51.09	74.19	53.14	53.88	40.25
DNA_Ginger	1.15	1.81	2.15	1.69	7.73	37.37	14.45	14.83	10.15

Tab. S7. Average CHH-DNA methylation levels within a given genomic feature and tissue type (as shown in Figure 2B, only autosomes). Abbreviations: arch: archegonia, anth: antherozoids, sporo: sporophyte, US/DS: upstream/downstream.

genomic feature	female apical notch	male apical notch	female thallus	male thallus	arch	anth	early sporo.	late sporo.	average across tissues
5' US, 2kb	0.67	1.03	1.31	1.36	4.47	25.67	6.49	8.87	6.24
5' UTR	0.50	0.84	0.98	1.25	3.76	26.28	3.02	4.16	5.10
exon	0.58	0.95	1.18	1.32	5.19	27.54	5.67	6.78	6.15
intron	0.49	0.83	0.90	1.07	3.79	23.13	3.19	4.67	4.76
3' UTR	0.49	0.84	0.89	1.06	4.07	27.96	2.57	3.86	5.22
3' DS, 2kb	0.64	1.01	1.30	1.34	4.64	27.24	6.30	8.73	6.40
intergenic	0.56	0.93	0.98	1.08	3.18	22.10	4.26	6.81	4.99
snRNA	0.72	1.10	1.41	1.45	4.86	26.02	8.55	12.48	7.07
rRNA	0.54	0.86	0.92	1.07	3.61	21.66	5.44	8.01	5.26
satellite	0.53	0.90	0.89	0.99	2.59	12.75	4.80	7.82	3.91
satellite_telo.	2.84	3.60	5.90	5.39	13.13	39.97	27.63	38.45	17.11
simple_repeat	2.05	2.81	4.84	5.12	12.15	36.00	25.37	34.24	15.32
uknRepeats	1.26	1.71	2.92	2.56	7.56	28.96	14.91	21.09	10.12
SINE	0.51	0.93	1.01	1.13	3.81	25.01	5.69	7.80	5.74
LINE_I	2.75	3.21	6.30	6.25	14.50	28.20	29.78	40.48	16.44
LINE_L1	3.03	3.75	6.60	6.25	15.29	39.33	27.49	35.71	17.18
LINE_L2	1.32	1.73	2.18	2.28	8.20	39.41	17.10	25.60	12.23
LINE_RTE	1.53	2.01	3.46	3.27	9.59	33.38	21.18	30.03	13.06
LINE_CRE	1.91	2.47	3.74	3.41	11.30	41.61	23.73	31.41	14.95
LINE_Tad1	0.52	0.86	1.03	1.05	4.01	23.37	5.47	7.89	5.53
RC_Helitron	1.13	1.58	1.96	2.28	7.42	36.43	17.89	25.34	11.75
LTR	2.33	3.47	5.36	6.23	12.10	37.87	25.17	34.69	15.90
LTR_Gypsy	2.70	3.39	6.45	5.92	12.59	36.34	24.37	34.83	15.82
LTR_Copia	1.58	2.13	3.27	2.94	8.57	35.32	19.44	28.69	12.74
LTR_ERV1	0.71	1.01	1.50	1.60	5.29	24.70	11.84	21.24	8.49
LTR_ERV4	0.64	0.87	1.10	1.15	3.74	21.89	7.67	14.89	6.49
LTR_Pao	0.47	0.79	0.97	1.16	4.08	21.42	5.59	7.97	5.31
LTR_DIRS	0.47	0.92	0.90	1.34	3.10	23.35	5.11	13.40	6.07
DNA	1.61	2.18	3.19	3.15	9.87	38.84	21.20	30.73	13.85
DNA_Sola	3.89	4.61	8.36	9.02	17.86	38.24	30.68	40.94	19.20
DNA_CMC	2.27	2.83	5.20	4.14	12.55	31.42	24.46	33.17	14.50
DNA_PIF/ Harbinger	2.21	2.78	4.38	4.41	12.56	38.39	25.77	34.28	15.60
DNA_MuLE	1.95	2.50	4.12	4.02	11.38	37.83	23.33	31.68	14.60
DNA_TcMar	1.54	2.11	2.99	2.74	9.13	38.87	21.27	29.92	13.57
DNA_hAT	1.80	2.42	3.54	3.35	10.42	39.89	22.30	30.55	14.28
DNA_Ginger	0.53	0.80	0.90	0.87	3.33	16.54	6.88	9.09	4.87

Tab. S8. Results of an analysis of deviance (i.e., ANOVA with deviances instead of variances) for the average DNA methylation in dependence of the sequence context, genomic feature context, tissue type, and all their interactions. Abbreviations: Df: degrees of freedom, DVC: deviance change, MDVC: mean deviance change, F: variance ratio , P: probability of type-I error, percDVC: percent of deviance change.

source of variation	Df	DVC	MDVC	F	P	percDVC
sequence context (SC)	2	257.49	128.75	17058.5	<10e-200	25.87
tissue type (TT)	7	212.42	30.35	4020.7	<10e-200	21.34
feature context (FC)	35	439.06	12.54	1662.1	<10e-200	44.12
SC:TT	14	26.91	1.92	254.7	<10e-200	2.70
SC:FC	70	24.46	0.35	46.3	<10e-200	2.46
TT:FC	245	19.24	0.08	10.4	<10e-200	1.93
SC:TT:FC	490	2.63	0.0054	0.7	>0.99999	0.26
Residuals	1728	12.98	0.0075			1.30

Tab. S9. Average DNA methylation levels in percent within a given sequence context and tissue type (as shown in Figure 3A, only sex-chromosomes).

	CpG	CHG	CHH	average across contexts
female apical notch (X)	72.99	32.63	2.31	35.97
male apical notch (Y)	74.70	33.69	2.53	36.97
female thallus (X)	75.24	41.95	5.12	40.77
male thallus (Y)	62.30	31.64	3.68	32.54
archegonia (X)	89.05	68.43	11.6	56.36
antherozoids (Y)	93.07	78.50	33.16	68.24
early sporophyte (X)	84.49	69.03	22.79	58.77
early sporophyte (Y)	80.71	61.77	22.64	55.04
late sporophyte (X)	84.77	69.78	32.79	62.45
late sporophyte (Y)	84.70	67.43	32.18	61.44
average across tissues	80.20	55.48	16.88	

Tab. S10. Number of differentially methylated cytosines (DMCs; FDR <= 0.001) found in pairwise comparisons between different individuals using the tissue types "apical notch", "thallus", and "gametes" as pseudo-replicates.

comparison	#CG-DMC	#CHG-DMC	#CHH-DMC
ind_2 *vs* ind_9	101	9	1
ind_2 *vs* ind_11	167	9	4
ind_2 *vs* ind_7	247	1	0
ind_2 *vs* ind_13	760	8	11
ind_2 *vs* ind_14	556	2	5
ind_9 *vs* ind_11	66	3	0
ind_9 *vs* ind_7	146	3	3
ind_9 *vs* ind_13	615	10	9
ind_9 *vs* ind_14	455	15	8
ind_11 *vs* ind_7	163	2	1
ind_11 *vs* ind_13	597	7	10
ind_11 *vs* ind_14	454	9	5
ind_7 *vs* ind_13	398	3	6
ind_7 *vs* ind_14	274	1	4
ind_13 *vs* ind_14	602	5	4
significant on average	373	6	5
total number tested	119563	122006	764095

Tab. S11. Enrichment analysis for DMCs with a gain in DNA methylation in the early sporophyte compared to the gametes ("set2") and DMCs with a gain in DNA methylation during sporophyte development ("set3") in the CG sequence context. Genomic features with less than 10 DMCs in total were not tested ("NA") for enrichment. The remaining features were tested with a two-sided Fisher test and P-values were adjusted for multiple testing (FDR, false discovery rate).

feature context	#CG-DMC set2	#CG-DMC set3	set2-set3 (percent difference)	FDR
5' US, 2kb	2	27	-9.12	1.4e-05
5' UTR	0	0	NA	NA
exon	7	12	-1.8	6.7e-01
intron	4	16	-4.36	2.7e-02
3' UTR	0	9	NA	NA
3' DS, 2kb	1	9	-2.92	4.7e-02
intergenic	1	6	NA	NA
snRNA	0	0	NA	NA
rRNA	0	0	NA	NA
satellite	219	0	80.81	1.2e-51
satellite_telo.	0	0	NA	NA
simple_repeat	0	0	NA	NA
uknRepeats	13	102	-32.43	9.0e-15
SINE	0	0	NA	NA
LINE_I	4	6	-0.71	1.0e+00
LINE_L1	0	0	NA	NA
LINE_L2	0	0	NA	NA
LINE_RTE	6	7	-0.34	1.0e+00
LINE_CRE	0	0	NA	NA
LINE_Tad1	0	0	NA	NA
RC_Helitron	0	0	NA	NA
LTR	0	0	NA	NA
LTR_Gypsy	6	17	-3.99	7.1e-02
LTR_Copia	4	29	-9.11	6.8e-05
LTR_ERV1	0	15	-5.47	2.1e-04
LTR_ERV4	0	2	NA	NA
LTR_Pao	0	0	NA	NA
LTR_DIRS	0	0	NA	NA
DNA	0	12	-4.38	1.5e-03
DNA_Sola	0	0	NA	NA
DNA_CMC	0	4	NA	NA
DNA_PIF/ Harbinger	0	0	NA	NA
DNA_MuLE	0	0	NA	NA
DNA_TcMar	0	0	NA	NA
DNA_hAT	3	3	NA	NA
DNA_Ginger	0	0	NA	NA
total counts	270	276		

Tab. S12. Enrichment analysis for DMCs with a gain in DNA methylation in the early sporophyte compared to the gametes ("set2") and DMCs with a gain in DNA methylation during sporophyte development ("set3") in the CHG sequence context. Genomic features with less than 10 DMCs in total were not tested ("NA") for enrichment. The remaining features were tested with a two-sided Fisher test and *P*-values were adjusted for multiple testing (FDR, false discovery rate).

feature context	#CHG-DMC set2	#CHG-DMC set3	set2-set3 (percent difference)	FDR
5' US, 2kb	59	74	-3.63	6.4e-04
5' UTR	4	0	NA	NA
exon	9	10	-0.42	4.9e-01
intron	24	30	-1.47	3.9e-02
3' UTR	0	9	NA	NA
3' DS, 2kb	46	52	-2.26	1.6e-02
intergenic	17	16	-0.51	5.6e-01
snRNA	0	1	NA	NA
rRNA	0	0	NA	NA
satellite	29	0	1.85	4.0e-06
satellite_telo.	1	0	NA	NA
simple_repeat	0	0	NA	NA
uknRepeats	442	427	-14.45	1.0e-06
SINE	0	1	NA	NA
LINE_I	168	10	9.72	1.1e-22
LINE_L1	27	5	1.22	1.6e-02
LINE_L2	0	0	NA	NA
LINE_RTE	83	29	2.4	1.5e-02
LINE_CRE	5	1	NA	NA
LINE_Tad1	6	0	NA	NA
RC_Helitron	1	0	NA	NA
LTR	2	0	NA	NA
LTR_Gypsy	364	49	18.33	2.3e-29
LTR_Copia	141	151	-6.09	1.2e-04
LTR_ERV1	21	34	-2.06	2.4e-03
LTR_ERV4	1	6	NA	NA
LTR_Pao	0	0	NA	NA
LTR_DIRS	0	0	NA	NA
DNA	9	70	-6.42	1.9e-18
DNA_Sola	3	0	NA	NA
DNA_CMC	53	7	2.68	2.3e-05
DNA_PIF/ Harbinger	2	0	NA	NA
DNA_MuLE	3	2	NA	NA
DNA_TcMar	20	5	0.78	1.3e-01
DNA_hAT	25	12	0.4	9.3e-01
DNA_Ginger	1	0	NA	NA
total counts	1566	1001		

Tab. S13. Enrichment analysis for DMCs with a gain in DNA methylation in the early sporophyte compared to the gametes ("set2") and DMCs with a gain in DNA methylation during sporophyte development ("set3") in the CHH sequence context. Genomic features with less than 10 DMCs in total were not tested ("NA") for enrichment. The remaining features were tested with a two-sided Fisher test and *P*-values were adjusted for multiple testing (FDR, false discovery rate).

feature context	#CHH-DMC set2	#CHH-DMC set3	set2-set3 (percent difference)	FDR
5' US, 2kb	749	515	-0.24	4.9e-01
5' UTR	47	3	0.19	7.3e-07
exon	91	32	0.19	8.8e-03
intron	124	160	-0.56	4.7e-08
3' UTR	17	37	-0.18	6.8e-05
3' DS, 2kb	476	459	-1.07	8.3e-09
intergenic	101	265	-1.4	2.0e-36
snRNA	36	24	-0.01	1.0e+00
rRNA	12	1	0.05	4.8e-02
satellite	8	2	0.02	6.4e-01
satellite_telo.	31	8	0.08	4.7e-02
simple_repeat	37	5	0.13	6.4e-04
uknRepeats	3885	5889	-23.78	3.6e-303
SINE	5	10	-0.05	7.5e-02
LINE_I	1280	127	4.87	3.6e-138
LINE_L1	389	125	0.87	1.7e-11
LINE_L2	22	14	0	1.0e+00
LINE_RTE	1535	572	2.9	5.0e-28
LINE_CRE	19	2	0.07	1.6e-02
LINE_Tad1	12	0	0.05	1.4e-02
RC_Helitron	52	14	0.14	7.3e-03
LTR	18	2	0.07	2.4e-02
LTR_Gypsy	7330	1926	19.47	4.5e-251
LTR_Copia	3770	2626	-1.44	9.1e-03
LTR_ERV1	136	158	-0.5	2.8e-06
LTR_ERV4	9	92	-0.6	9.8e-27
LTR_Pao	2	0	NA	NA
LTR_DIRS	0	0	NA	NA
DNA	158	489	-2.72	1.8e-76
DNA_Sola	72	9	0.26	2.1e-07
DNA_CMC	498	85	1.64	1.2e-36
DNA_PIF/ Harbinger	86	19	0.25	2.2e-05
DNA_MuLE	221	73	0.48	2.0e-06
DNA_TcMar	294	131	0.4	1.5e-03
DNA_hAT	709	402	0.37	1.1e-01
DNA_Ginger	4	0	NA	NA
total counts	22235	14276		

Tab. S14. RNA concentration measurements and total RNA input material used for the reverse transcription reaction for the Droplet Digital PCR (ddPCR) assay.

condition	replicate	RNA input (ng)
female apical notch	1	864
male apical notch	1	510.3
female thallus	1	497.7
male thallus	1	353
archegonia	1	145
antheridia	1	4.8575
early sporophyte	1	288.55
late sporophyte	1	747.2
female apical notch	2	876
male apical notch	2	620
female thallus	2	420
male thallus	2	323
archegonia	2	71.05
antheridia	2	2.523
early sporophyte	2	413
late sporophyte	2	387.1
female apical notch	3	828
male apical notch	3	660
female thallus	3	353
male thallus	3	347
archegonia	3	49.59
antheridia	3	1.6095
early sporophyte	3	447
late sporophyte	3	607

Tab. S15. Primer sequences used in the Droplet Digital PCR (ddPCR) assay.

gene ID	gene name	orientation	sequence (5' to 3')	reference	intron spanning
Mapoly0100s0027	Mp*APT3*	forward	CGAAAGCCCAAGAAGCTACC	[2]	yes
		reverse	GTACCCCCGGTTGCAATAAG		
Mapoly0016s0139	Mp*ACT7*	forward	AGGCATCTGGTATCCACGAG	[2]	no
		reverse	ACATGGTCGTTCCTCCAGAC		
Mapoly0038s0027.1	Mp*MET*	forward	GATCACCGATCCTCAGCCCA	this study	yes
		reverse	GAAATCCCTGAGAGCGGGCA		
Mapoly0060s0056.1	Mp*CMTa*	forward	GTCGGTGAAGGAGAGGTACA	this study	yes
		reverse	GGGAAGCTGCATCAAAGC		
Mapoly0091s0051.1	Mp*CMTb*	forward	CTCATGGTCAGCGCCTTT	this study	yes
		reverse	TACAGACACAGCGTTGCC		
Mapoly0103s0053.1	Mp*DRMa*	forward	GAGAACGGCAAGGAGGGATT	this study	yes
		reverse	CCCGTTCGGTTTCTACGGAT		
Mapoly0109s0015.1	Mp*DRMb*	forward	GGACCATGAAACCTTGCAG	this study	no
		reverse	AGAAAATCGGTTCTTGGCAG		

Tab. S16. Concentrations and efficiencies of primers used in the Droplet Digital PCR (ddPCR) assay. All primers were tested across a dilution series ranging from undiluted cDNA (cDNA was generated from 300 ng total RNA) to a 0.00005 dilution (corresponding to a theoretical minimal input of 0.115 pg cDNA). The amplification efficiency for the primers targeting Mp*CMTb* decreased in dilutions above 0.0005. Thus, two efficiency values are indicated for these primers excluding/including dilutions above 0.0005, respectively.

target	optimal concentration	efficiency	R^2
Mp*MET*	200 nM	109.90%	0.993
Mp*CMTa*	150 nM	118.50%	0.98
Mp*CMTb*	150 nM	96.2%/90.485%	0.999/0.996
Mp*DRMa*	200 nM	111.50%	0.982
Mp*DRMb*	200 nM	100.70%	0.998

Tab. S17. Results from the Droplet Digital PCR (ddPCR) assay. "gene/ref, RT+/RT-" refer to expression values of test genes and reference genes from the reaction using the cDNA libraries (RT+) or a mock library in which there was no cDNA (RT-).

condition	gene	reference gene	gene, RT+	gene, RT-	reference, RT+	reference, RT-
female apical notch	Mp*MET*	Mp*APT3*	19.6	NA	260	NA
male apical notch	Mp*MET*	Mp*APT3*	10.6	NA	1.9	NA
female thallus	Mp*MET*	Mp*APT3*	8	NA	461	NA
male thallus	Mp*MET*	Mp*APT3*	0.45	NA	13.7	NA
archegonia	Mp*MET*	Mp*APT3*	0.6	0	1.35	NA
antherozoids	Mp*MET*	Mp*APT3*	4.3	0	0	NA
early sporophyte	Mp*MET*	Mp*APT3*	13.4	0.06	55	NA
late sporophyte	Mp*MET*	Mp*APT3*	4.1	0	2.7	NA
female apical notch	Mp*MET*	Mp*APT3*	18.3	NA	41.3	NA
male apical notch	Mp*MET*	Mp*APT3*	313	NA	2620	NA
female thallus	Mp*MET*	Mp*APT3*	0.66	NA	11.5	NA
male thallus	Mp*MET*	Mp*APT3*	15.9	NA	525	NA
archegonia	Mp*MET*	Mp*APT3*	0.38	NA	1.9	NA
antherozoids	Mp*MET*	Mp*APT3*	7.5	NA	0.32	NA
early sporophyte	Mp*MET*	Mp*APT3*	0.51	NA	0.64	NA
late sporophyte	Mp*MET*	Mp*APT3*	3.9	NA	27.2	NA
female apical notch	Mp*MET*	Mp*APT3*	21.8	NA	49.1	NA
male apical notch	Mp*MET*	Mp*APT3*	13.9	NA	45.4	NA
female thallus	Mp*MET*	Mp*APT3*	18.6	NA	384	NA
male thallus	Mp*MET*	Mp*APT3*	2.1	NA	34.9	NA
archegonia	Mp*MET*	Mp*APT3*	0.98	NA	3.1	NA
antherozoids	Mp*MET*	Mp*APT3*	6.6	NA	0.24	NA
early sporophyte	Mp*MET*	Mp*APT3*	26.1	NA	75.3	NA
late sporophyte	Mp*MET*	Mp*APT3*	36.7	NA	286	NA
female apical notch	Mp*CMTa*	Mp*APT3*	19.4	NA	260	NA
male apical notch	Mp*CMTa*	Mp*APT3*	1.33	NA	1.9	NA
female thallus	Mp*CMTa*	Mp*APT3*	16.2	NA	461	NA
male thallus	Mp*CMTa*	Mp*APT3*	0.28	NA	13.7	NA
archegonia	Mp*CMTa*	Mp*APT3*	0.19	NA	1.35	NA
antherozoids	Mp*CMTa*	Mp*APT3*	0.06	NA	0	NA
early sporophyte	Mp*CMTa*	Mp*APT3*	64.5	NA	55	NA
late sporophyte	Mp*CMTa*	Mp*APT3*	0.5	NA	2.7	NA
female apical notch	Mp*CMTa*	Mp*APT3*	6.1	NA	41.3	NA
male apical notch	Mp*CMTa*	Mp*APT3*	290	NA	2620	NA
female thallus	Mp*CMTa*	Mp*APT3*	0.59	NA	11.5	NA
male thallus	Mp*CMTa*	Mp*APT3*	13.8	NA	525	NA
archegonia	Mp*CMTa*	Mp*APT3*	0.06	NA	1.9	NA
antherozoids	Mp*CMTa*	Mp*APT3*	0.06	NA	0.32	NA
early sporophyte	Mp*CMTa*	Mp*APT3*	0.83	NA	0.64	NA
late sporophyte	Mp*CMTa*	Mp*APT3*	5.1	NA	27.2	NA
female apical notch	Mp*CMTa*	Mp*APT3*	8.6	NA	49.1	NA
male apical notch	Mp*CMTa*	Mp*APT3*	10	NA	45.4	NA

condition	gene	reference gene	gene, RT+	gene, RT-	reference, RT+	reference, RT-
female thallus	Mp*CMTa*	Mp*APT3*	14.5	NA	384	NA
male thallus	Mp*CMTa*	Mp*APT3*	1.3	NA	34.9	NA
archegonia	Mp*CMTa*	Mp*APT3*	0.6	NA	3.1	NA
antherozoids	Mp*CMTa*	Mp*APT3*	0	NA	0.24	NA
early sporophyte	Mp*CMTa*	Mp*APT3*	107.4	NA	75.3	NA
late sporophyte	Mp*CMTa*	Mp*APT3*	48.5	NA	286	NA
female apical notch	Mp*CMTb*	Mp*APT3*	0.06	NA	260	NA
male apical notch	Mp*CMTb*	Mp*APT3*	0	NA	1.9	NA
female thallus	Mp*CMTb*	Mp*APT3*	0	NA	461	NA
male thallus	Mp*CMTb*	Mp*APT3*	0	NA	13.7	NA
archegonia	Mp*CMTb*	Mp*APT3*	0.11	NA	1.35	NA
antherozoids	Mp*CMTb*	Mp*APT3*	0	NA	0	NA
early sporophyte	Mp*CMTb*	Mp*APT3*	347	NA	55	NA
late sporophyte	Mp*CMTb*	Mp*APT3*	0.06	NA	2.7	NA
female apical notch	Mp*CMTb*	Mp*APT3*	0.2	NA	41.3	NA
male apical notch	Mp*CMTb*	Mp*APT3*	0.7	NA	2620	NA
female thallus	Mp*CMTb*	Mp*APT3*	0	NA	11.5	NA
male thallus	Mp*CMTb*	Mp*APT3*	0	NA	525	NA
archegonia	Mp*CMTb*	Mp*APT3*	0	NA	1.9	NA
antherozoids	Mp*CMTb*	Mp*APT3*	0	NA	0.32	NA
early sporophyte	Mp*CMTb*	Mp*APT3*	9	NA	0.64	NA
late sporophyte	Mp*CMTb*	Mp*APT3*	0.33	NA	27.2	NA
female apical notch	Mp*CMTb*	Mp*APT3*	0	NA	49.1	NA
male apical notch	Mp*CMTb*	Mp*APT3*	0	NA	45.4	NA
female thallus	Mp*CMTb*	Mp*APT3*	0.06	NA	384	NA
male thallus	Mp*CMTb*	Mp*APT3*	0	NA	34.9	NA
archegonia	Mp*CMTb*	Mp*APT3*	0	NA	3.1	NA
antherozoids	Mp*CMTb*	Mp*APT3*	0.19	NA	0.24	NA
early sporophyte	Mp*CMTb*	Mp*APT3*	373	NA	75.3	NA
late sporophyte	Mp*CMTb*	Mp*APT3*	4.5	NA	286	NA
female apical notch	Mp*DRMa*	Mp*APT3*	1.14	NA	260	NA
male apical notch	Mp*DRMa*	Mp*APT3*	1.5	NA	1.9	NA
female thallus	Mp*DRMa*	Mp*APT3*	3.6	NA	461	NA
male thallus	Mp*DRMa*	Mp*APT3*	0.23	NA	13.7	NA
archegonia	Mp*DRMa*	Mp*APT3*	0.42	NA	1.35	NA
antherozoids	Mp*DRMa*	Mp*APT3*	0.82	NA	0	NA
early sporophyte	Mp*DRMa*	Mp*APT3*	245	NA	55	NA
late sporophyte	Mp*DRMa*	Mp*APT3*	0.63	NA	2.7	NA
female apical notch	Mp*DRMa*	Mp*APT3*	2.5	NA	41.3	NA
male apical notch	Mp*DRMa*	Mp*APT3*	46.4	NA	2620	NA
female thallus	Mp*DRMa*	Mp*APT3*	0	NA	11.5	NA
male thallus	Mp*DRMa*	Mp*APT3*	4.8	NA	525	NA
archegonia	Mp*DRMa*	Mp*APT3*	0.25	NA	1.9	NA
antherozoids	Mp*DRMa*	Mp*APT3*	2	NA	0.32	NA
early sporophyte	Mp*DRMa*	Mp*APT3*	1.45	NA	0.64	NA
late sporophyte	Mp*DRMa*	Mp*APT3*	1.29	NA	27.2	NA

condition	gene	reference gene	gene, RT+	gene, RT-	reference, RT+	reference, RT-
female apical notch	Mp*DRMa*	Mp*APT3*	1.3	NA	49.1	NA
male apical notch	Mp*DRMa*	Mp*APT3*	1.09	NA	45.4	NA
female thallus	Mp*DRMa*	Mp*APT3*	2.2	NA	384	NA
male thallus	Mp*DRMa*	Mp*APT3*	0.74	NA	34.9	NA
archegonia	Mp*DRMa*	Mp*APT3*	0.63	NA	3.1	NA
antherozoids	Mp*DRMa*	Mp*APT3*	0.87	NA	0.24	NA
early sporophyte	Mp*DRMa*	Mp*APT3*	125.8	NA	75.3	NA
late sporophyte	Mp*DRMa*	Mp*APT3*	18.6	NA	286	NA
female apical notch	Mp*DRMb*	Mp*APT3*	4.5	6	260	NA
male apical notch	Mp*DRMb*	Mp*APT3*	6.7	8.7	1.9	NA
female thallus	Mp*DRMb*	Mp*APT3*	1.14	1.33	461	NA
male thallus	Mp*DRMb*	Mp*APT3*	0	0	13.7	NA
archegonia	Mp*DRMb*	Mp*APT3*	8.5	0	1.35	NA
antherozoids	Mp*DRMb*	Mp*APT3*	0.5	0.79	0	NA
early sporophyte	Mp*DRMb*	Mp*APT3*	680.6	0.19	55	NA
late sporophyte	Mp*DRMb*	Mp*APT3*	3.9	2.1	2.7	NA
female apical notch	Mp*DRMb*	Mp*APT3*	6.8	97	41.3	NA
male apical notch	Mp*DRMb*	Mp*APT3*	16	21.4	2620	NA
female thallus	Mp*DRMb*	Mp*APT3*	0.28	0.27	11.5	NA
male thallus	Mp*DRMb*	Mp*APT3*	0.28	0.29	525	NA
archegonia	Mp*DRMb*	Mp*APT3*	4.96	0	1.9	NA
antherozoids	Mp*DRMb*	Mp*APT3*	4.52	2.6	0.32	NA
early sporophyte	Mp*DRMb*	Mp*APT3*	24	0.12	0.64	NA
late sporophyte	Mp*DRMb*	Mp*APT3*	0.84	0.12	27.2	NA
female apical notch	Mp*DRMb*	Mp*APT3*	4.6	4.1	49.1	NA
male apical notch	Mp*DRMb*	Mp*APT3*	1.6	1.17	45.4	NA
female thallus	Mp*DRMb*	Mp*APT3*	0.37	0.31	384	NA
male thallus	Mp*DRMb*	Mp*APT3*	0.6	0.63	34.9	NA
archegonia	Mp*DRMb*	Mp*APT3*	10.5	0	3.1	NA
antherozoids	Mp*DRMb*	Mp*APT3*	5.9	1.48	0.24	NA
early sporophyte	Mp*DRMb*	Mp*APT3*	774	0	75.3	NA
late sporophyte	Mp*DRMb*	Mp*APT3*	14.9	1.42	286	NA
female apical notch	Mp*MET*	Mp*ACT7*	19.6	NA	1234	4.8
male apical notch	Mp*MET*	Mp*ACT7*	10.6	NA	11.5	6.7
female thallus	Mp*MET*	Mp*ACT7*	8	NA	4400	0.7
male thallus	Mp*MET*	Mp*ACT7*	0.45	NA	76.9	0
archegonia	Mp*MET*	Mp*ACT7*	0.6	0	0.84	0
antherozoids	Mp*MET*	Mp*ACT7*	4.3	0	0.44	0.13
early sporophyte	Mp*MET*	Mp*ACT7*	13.4	0.06	951	0
late sporophyte	Mp*MET*	Mp*ACT7*	4.1	0	19.8	0.54
female apical notch	Mp*MET*	Mp*ACT7*	18.3	NA	122.2	9.6
male apical notch	Mp*MET*	Mp*ACT7*	313	NA	12540	16.9
female thallus	Mp*MET*	Mp*ACT7*	0.66	NA	47.8	0.21
male thallus	Mp*MET*	Mp*ACT7*	15.9	NA	1710	0.06
archegonia	Mp*MET*	Mp*ACT7*	0.38	NA	3.8	0

condition	gene	reference gene	gene, RT+	gene, RT-	reference, RT+	reference, RT-
antherozoids	Mp*MET*	Mp*ACT7*	7.5	NA	4.2	1.8
early sporophyte	Mp*MET*	Mp*ACT7*	0.51	NA	8.4	0
late sporophyte	Mp*MET*	Mp*ACT7*	3.9	NA	79.8	0.37
female apical notch	Mp*MET*	Mp*ACT7*	21.8	NA	127.5	4.2
male apical notch	Mp*MET*	Mp*ACT7*	13.9	NA	164.5	1.5
female thallus	Mp*MET*	Mp*ACT7*	18.6	NA	1512	0.33
male thallus	Mp*MET*	Mp*ACT7*	2.1	NA	221	0.24
archegonia	Mp*MET*	Mp*ACT7*	0.98	NA	9.9	0
antherozoids	Mp*MET*	Mp*ACT7*	6.6	NA	4	1.24
early sporophyte	Mp*MET*	Mp*ACT7*	26.1	NA	649	0
late sporophyte	Mp*MET*	Mp*ACT7*	36.7	NA	859	1.7
female apical notch	Mp*CMTa*	Mp*ACT7*	19.4	NA	1234	4.8
male apical notch	Mp*CMTa*	Mp*ACT7*	1.33	NA	11.5	6.7
female thallus	Mp*CMTa*	Mp*ACT7*	16.2	NA	4400	0.7
male thallus	Mp*CMTa*	Mp*ACT7*	0.28	NA	76.9	0
archegonia	Mp*CMTa*	Mp*ACT7*	0.19	NA	0.84	0
antherozoids	Mp*CMTa*	Mp*ACT7*	0.06	NA	0.44	0.13
early sporophyte	Mp*CMTa*	Mp*ACT7*	64.5	NA	951	0
late sporophyte	Mp*CMTa*	Mp*ACT7*	0.5	NA	19.8	0.54
female apical notch	Mp*CMTa*	Mp*ACT7*	6.1	NA	122.2	9.6
male apical notch	Mp*CMTa*	Mp*ACT7*	290	NA	12540	16.9
female thallus	Mp*CMTa*	Mp*ACT7*	0.59	NA	47.8	0.21
male thallus	Mp*CMTa*	Mp*ACT7*	13.8	NA	1710	0.06
archegonia	Mp*CMTa*	Mp*ACT7*	0.06	NA	3.8	0
antherozoids	Mp*CMTa*	Mp*ACT7*	0.06	NA	4.2	1.8
early sporophyte	Mp*CMTa*	Mp*ACT7*	0.83	NA	8.4	0
late sporophyte	Mp*CMTa*	Mp*ACT7*	5.1	NA	79.8	0.37
female apical notch	Mp*CMTa*	Mp*ACT7*	8.6	NA	127.5	4.2
male apical notch	Mp*CMTa*	Mp*ACT7*	10	NA	164.5	1.5
female thallus	Mp*CMTa*	Mp*ACT7*	14.5	NA	1512	0.33
male thallus	Mp*CMTa*	Mp*ACT7*	1.3	NA	221	0.24
archegonia	Mp*CMTa*	Mp*ACT7*	0.6	NA	9.9	0
antherozoids	Mp*CMTa*	Mp*ACT7*	0	NA	4	1.24
early sporophyte	Mp*CMTa*	Mp*ACT7*	107.4	NA	649	0
late sporophyte	Mp*CMTa*	Mp*ACT7*	48.5	NA	859	1.7
female apical notch	Mp*CMTb*	Mp*ACT7*	0.06	NA	1234	4.8
male apical notch	Mp*CMTb*	Mp*ACT7*	0	NA	11.5	6.7
female thallus	Mp*CMTb*	Mp*ACT7*	0	NA	4400	0.7
male thallus	Mp*CMTb*	Mp*ACT7*	0	NA	76.9	0
archegonia	Mp*CMTb*	Mp*ACT7*	0.11	NA	0.84	0
antherozoids	Mp*CMTb*	Mp*ACT7*	0	NA	0.44	0.13
early sporophyte	Mp*CMTb*	Mp*ACT7*	347	NA	951	0
late sporophyte	Mp*CMTb*	Mp*ACT7*	0.06	NA	19.8	0.54
female apical notch	Mp*CMTb*	Mp*ACT7*	0.2	NA	122.2	9.6
male apical notch	Mp*CMTb*	Mp*ACT7*	0.7	NA	12540	16.9

condition	gene	reference gene	gene, RT+	gene, RT-	reference, RT+	reference, RT-
female thallus	Mp*CMTb*	Mp*ACT7*	0	NA	47.8	0.21
male thallus	Mp*CMTb*	Mp*ACT7*	0	NA	1710	0.06
archegonia	Mp*CMTb*	Mp*ACT7*	0	NA	3.8	0
antherozoids	Mp*CMTb*	Mp*ACT7*	0	NA	4.2	1.8
early sporophyte	Mp*CMTb*	Mp*ACT7*	9	NA	8.4	0
late sporophyte	Mp*CMTb*	Mp*ACT7*	0.33	NA	79.8	0.37
female apical notch	Mp*CMTb*	Mp*ACT7*	0	NA	127.5	4.2
male apical notch	Mp*CMTb*	Mp*ACT7*	0	NA	164.5	1.5
female thallus	Mp*CMTb*	Mp*ACT7*	0.06	NA	1512	0.33
male thallus	Mp*CMTb*	Mp*ACT7*	0	NA	221	0.24
archegonia	Mp*CMTb*	Mp*ACT7*	0	NA	9.9	0
antherozoids	Mp*CMTb*	Mp*ACT7*	0.19	NA	4	1.24
early sporophyte	Mp*CMTb*	Mp*ACT7*	373	NA	649	0
late sporophyte	Mp*CMTb*	Mp*ACT7*	4.5	NA	859	1.7
female apical notch	Mp*DRMa*	Mp*ACT7*	1.14	NA	1234	4.8
male apical notch	Mp*DRMa*	Mp*ACT7*	1.5	NA	11.5	6.7
female thallus	Mp*DRMa*	Mp*ACT7*	3.6	NA	4400	0.7
male thallus	Mp*DRMa*	Mp*ACT7*	0.23	NA	76.9	0
archegonia	Mp*DRMa*	Mp*ACT7*	0.42	NA	0.84	0
antherozoids	Mp*DRMa*	Mp*ACT7*	0.82	NA	0.44	0.13
early sporophyte	Mp*DRMa*	Mp*ACT7*	245	NA	951	0
late sporophyte	Mp*DRMa*	Mp*ACT7*	0.63	NA	19.8	0.54
female apical notch	Mp*DRMa*	Mp*ACT7*	2.5	NA	122.2	9.6
male apical notch	Mp*DRMa*	Mp*ACT7*	46.4	NA	12540	16.9
female thallus	Mp*DRMa*	Mp*ACT7*	0	NA	47.8	0.21
male thallus	Mp*DRMa*	Mp*ACT7*	4.8	NA	1710	0.06
archegonia	Mp*DRMa*	Mp*ACT7*	0.25	NA	3.8	0
antherozoids	Mp*DRMa*	Mp*ACT7*	2	NA	4.2	1.8
early sporophyte	Mp*DRMa*	Mp*ACT7*	1.45	NA	8.4	0
late sporophyte	Mp*DRMa*	Mp*ACT7*	1.29	NA	79.8	0.37
female apical notch	Mp*DRMa*	Mp*ACT7*	1.3	NA	127.5	4.2
male apical notch	Mp*DRMa*	Mp*ACT7*	1.09	NA	164.5	1.5
female thallus	Mp*DRMa*	Mp*ACT7*	2.2	NA	1512	0.33
male thallus	Mp*DRMa*	Mp*ACT7*	0.74	NA	221	0.24
archegonia	Mp*DRMa*	Mp*ACT7*	0.63	NA	9.9	0
antherozoids	Mp*DRMa*	Mp*ACT7*	0.87	NA	4	1.24
early sporophyte	Mp*DRMa*	Mp*ACT7*	125.8	NA	649	0
late sporophyte	Mp*DRMa*	Mp*ACT7*	18.6	NA	859	1.7
female apical notch	Mp*DRMb*	Mp*ACT7*	4.5	6	1234	4.8
male apical notch	Mp*DRMb*	Mp*ACT7*	6.7	8.7	11.5	6.7
female thallus	Mp*DRMb*	Mp*ACT7*	1.14	1.33	4400	0.7
male thallus	Mp*DRMb*	Mp*ACT7*	0	0	76.9	0
archegonia	Mp*DRMb*	Mp*ACT7*	8.5	0	0.84	0
antherozoids	Mp*DRMb*	Mp*ACT7*	0.5	0.79	0.44	0.13
early sporophyte	Mp*DRMb*	Mp*ACT7*	680.6	0.19	951	0
late sporophyte	Mp*DRMb*	Mp*ACT7*	3.9	2.1	19.8	0.54

condition	gene	reference gene	gene, RT+	gene, RT-	reference, RT+	reference, RT-
female apical notch	Mp*DRMb*	Mp*ACT7*	6.8	97	122.2	9.6
male apical notch	Mp*DRMb*	Mp*ACT7*	16	21.4	12540	16.9
female thallus	Mp*DRMb*	Mp*ACT7*	0.28	0.27	47.8	0.21
male thallus	Mp*DRMb*	Mp*ACT7*	0.28	0.29	1710	0.06
archegonia	Mp*DRMb*	Mp*ACT7*	4.96	0	3.8	0
antherozoids	Mp*DRMb*	Mp*ACT7*	4.52	2.6	4.2	1.8
early sporophyte	Mp*DRMb*	Mp*ACT7*	24	0.12	8.4	0
late sporophyte	Mp*DRMb*	Mp*ACT7*	0.84	0.12	79.8	0.37
female apical notch	Mp*DRMb*	Mp*ACT7*	4.6	4.1	127.5	4.2
male apical notch	Mp*DRMb*	Mp*ACT7*	1.6	1.17	164.5	1.5
female thallus	Mp*DRMb*	Mp*ACT7*	0.37	0.31	1512	0.33
male thallus	Mp*DRMb*	Mp*ACT7*	0.6	0.63	221	0.24
archegonia	Mp*DRMb*	Mp*ACT7*	10.5	0	9.9	0
antherozoids	Mp*DRMb*	Mp*ACT7*	5.9	1.48	4	1.24
early sporophyte	Mp*DRMb*	Mp*ACT7*	774	0	649	0
late sporophyte	Mp*DRMb*	Mp*ACT7*	14.9	1.42	859	1.7

References

1. Bowman JL, Kohchi T, Yamato KT, Jenkins J, Shu S, *et. al.* Insights into land plant evolution garnered from the *Marchantia polymorpha* genome. Cell. 2017;171:287–304.

2. Saint-Marcoux D, Proust H, Dolan L, Langdale JA. Identification of reference genes for real-time quantitative PCR experiments in the liverwort *Marchantia polymorpha*. PLOS ONE. 2015;10:e0118678.
